# Planarian Anatomy Ontology: a resource to connect data within and across experimental platforms

**DOI:** 10.1242/dev.196097

**Published:** 2021-08-02

**Authors:** Stephanie H. Nowotarski, Erin L. Davies, Sofia M. C. Robb, Eric J. Ross, Nicolas Matentzoglu, Viraj Doddihal, Mol Mir, Melainia McClain, Alejandro Sánchez Alvarado

**Affiliations:** 1 Stowers Institute for Medical Research, Kansas City, MO 64110, USA; 2 Howard Hughes Medical Institute, Chevy Chase, MD 20815, USA; 3 Center for Cancer Research, National Cancer Institute, National Institutes of Health, Frederick, MD 21702, USA; 4 European Bioinformatics Institute (EMBL-EBI), European Molecular Biology Laboratory, Wellcome Genome Campus, Hinxton, Cambridge CB10 1SD, UK

**Keywords:** Planarian, Ontology, Anatomy, Staging, Embryogenesis, Regeneration

## Abstract

As the planarian research community expands, the need for an interoperable data organization framework for tool building has become increasingly apparent. Such software would streamline data annotation and enhance cross-platform and cross-species searchability. We created the Planarian Anatomy Ontology (PLANA), an extendable relational framework of defined *Schmidtea mediterranea* (*Smed*) anatomical terms used in the field. At publication, PLANA contains over 850 terms describing *Smed* anatomy from subcellular to system levels across all life cycle stages, in intact animals and regenerating body fragments. Terms from other anatomy ontologies were imported into PLANA to promote interoperability and comparative anatomy studies. To demonstrate the utility of PLANA as a tool for data curation, we created resources for planarian embryogenesis, including a staging series and molecular fate-mapping atlas, and the Planarian Anatomy Gene Expression database, which allows retrieval of a variety of published transcript/gene expression data associated with PLANA terms. As an open-source tool built using FAIR (findable, accessible, interoperable, reproducible) principles, our strategy for continued curation and versioning of PLANA also provides a platform for community-led growth and evolution of this resource.

## Abstract (precis)

**Summary:** Description of the construction of an anatomy ontology tool for planaria with examples of its potential use to curate and mine data across multiple experimental platforms.

## INTRODUCTION

Researchers using the free-living, freshwater planarian *Schmidtea mediterranea* (*Smed*) are rapidly generating genomic, transcriptomic, phenotypic and anatomical data. However, the field lacks tools to facilitate straightforward, reliable navigation and integration of data across experimental platforms and publications. *Smed* anatomical information has been garnered using techniques for structural and ultrastructural visualization (e.g. histological staining, and scanning and transmission electron microscopy), as well as molecular techniques that report gene expression or protein localization *in situ* (e.g. whole-mount *in situ* hybridization, immunohistochemistry and immunofluorescence). Gene discovery has been facilitated through sequenced *Smed* genome assemblies (Robb et al., 2008, 2015; Grohme et al., 2018) and *de novo* assembled *Smed* transcriptomes (Adamidi et al., 2011; Sandmann et al., 2011; Labbé et al., 2012; Rouhana et al., 2012; Srivastava et al., 2014; Tu et al., 2015; Brandl et al., 2016). Microarray analyses (Eisenhoffer et al., 2008; Wagner et al., 2012), and bulk (Blythe et al., 2010; Solana et al., 2012; Davies et al., 2017) and single-cell RNA sequencing (RNA-Seq) (Wurtzel et al., 2015; Fincher et al., 2018; Plass et al., 2018; Zeng et al., 2018) have identified cell type- and tissue-enriched biomarkers, as well as candidate genes implicated in biological processes of interest for functional interrogation via whole-animal RNAi knockdown (Sánchez et al., 1999; Newmark et al., 2003; Reddien et al., 2005). Efficient integration and synthesis of this massive and expanding trove of morphological, molecular and functional data requires tools built using common anatomical vocabulary and syntax, and, most importantly, a method of organization that allows data to be easily retrieved by any category.

Big data must be readable, reusable and extensible by humans and computers. Ontologies (Gruber, 1993) excel at this crosstalk, creating common understanding within a domain of knowledge by placing entities described in a controlled language in relationship to each other using either explicitly defined (asserted) or inferred statements. The resulting structure is a representation of knowledge, readable by both humans and machines, that is able to retrieve both asserted and inferred axioms, i.e. a knowledge graph. This structure has made ontologies ubiquitous in the digital age, whereby frameworks such as the semantic web facilitate information sharing across automated systems. Codifying knowledge using an ontological framework also promotes sharing data according to FAIR (findable, accessible, interoperable and reproducible) practices (Wilkinson et al., 2016).

Ontology structures can be used to explore, understand and discover relationships among data and to begin to develop testable hypotheses. The Gene Ontology (GO) project is a clear example of the utility of ontologies in biological sciences. GO is a well-known, highly used framework that endeavors to ascribe putative functions to genes across species based on sequence homology, from the molecular to the organismal level (Ashburner et al., 2000). The hierarchical organization in GO, defined by relationships between terms, facilitates refinement or expansion of candidate gene lists from gene expression studies. For example, a gene list associated with pigmentation can be refined by selecting a more granular category, such as cellular pigmentation. GO is used in tools that provide a first-pass analysis of molecular and cellular processes most likely to be enriched or perturbed between experimental samples.

Anatomy ontologies are also widely used, inherently didactic resources that have been developed for many research organisms, including slime molds (Gaudet et al., 2008), nematodes (Lee and Sternberg, 2003), fruit flies (Costa et al., 2013), frogs (Segerdell et al., 2008), zebrafish (Van Slyke et al., 2014), mice (Hayamizu et al., 2013) and human (Bard, 2012). These ontologies are integral for the annotation of field resources such as Virtual Fly Brain (Milyaev et al., 2012; Osumi-Sutherland et al., 2014). In addition to providing a controlled vocabulary and means of streamlining data annotation, these frameworks also facilitate comparative studies on animal development and evolution. One way this is accomplished is by making species-specific ontologies compatible and interoperable with Uberon, a cross-species gross anatomy ontology (Mungall et al., 2012). The interoperability of ontologies enriches and extends navigation among disparate datasets. Interoperability of ontologies is provided through parallel data structures and common annotations allowing systems to mutually exchange and make use of information. For example, it will soon be possible to identify evolutionarily conserved genes required for ciliogenesis, along with genes expressed in cilia, via searches that use GO, Uberon, and species-specific anatomy and phenotype ontologies. To maximize the scientific utility and visibility of big data generated by the planarian research community, the field requires new bioinformatic tools built using ontologies that will improve data archival practices and search functions across different experimental platforms. Here, we debut the Planarian Anatomy Ontology (PLANA) as an organizational framework and demonstrate its utility when integrated into tools for data annotation and integration across 155 published data sets.

## RESULTS AND DISCUSSION

### Annotating anatomical terms: classes

PLANA is an extendable framework of defined terms that aims to describe *Smed* anatomy holistically across all life cycle stages for asexually and sexually reproducing biotypes. To ensure that PLANA encompasses all *Smed* anatomical terms used in the field, we conducted a review of 200 primary research citations from 2005 to 2019 (Table S1) and identified 658 terms pertaining to biotypes, life cycle stages, embryonic, adult and regenerating anatomical structures, subcellular components, cells, tissues, organs, anatomical systems, body regions, anatomical spaces (e.g. cavities and lumens), anatomical surfaces, boundaries, planes and axes. Of these 658 terms, 380 were synonymous (e.g. eye and photoreceptor), resulting in a final set of 278 terms commonly used by the planarian community. Hereafter, we call these terms classes. Each class has a primary name (label) and may have supporting synonym(s). In addition, classes were imported from other ontologies and composite classes (described below) were created. In all, PLANA version v2021-04-05 has a final class count of 863.

**Fig. 1.**
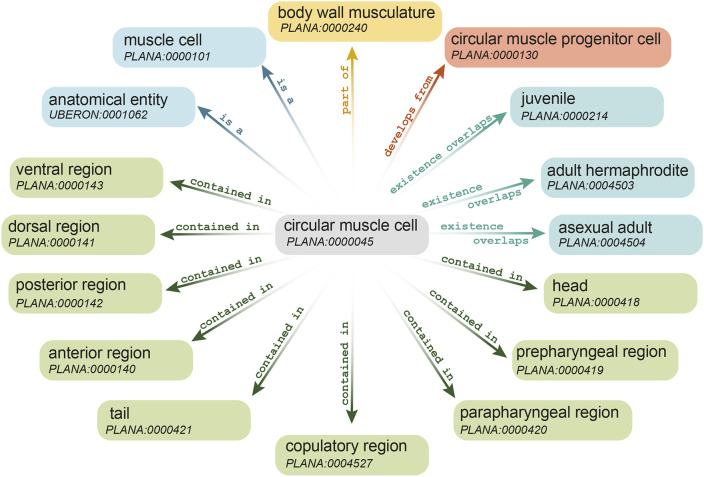
**PLANA classes are linked to one another through relationship terms.** The anatomical class ‘circular muscle cell’ is shown in relation to other PLANA classes. Different colors reflect the different types of relationships between the classes. Relationships shown are is a (blue), part of (yellow), develops from (salmon), existence overlaps (aqua) and contained in (green).

Although a list of anatomical terms is useful, the strength of an ontology derives from the ability to annotate classes with metadata and to organize classes hierarchically into a relational network. Each class has its own set of categorical, spatial, temporal and developmental relationships to other classes (Fig. 1). Following the convention set forth by Van Slyke et al. (2014), classes are represented using single quotation marks, and, although their identification number (ID) generally follows (e.g. ‘epidermis’ PLANA:0000034), we omit the ID here for readability. All IDs for PLANA classes mentioned in the text are found in Table S2.

**Fig. 2.**
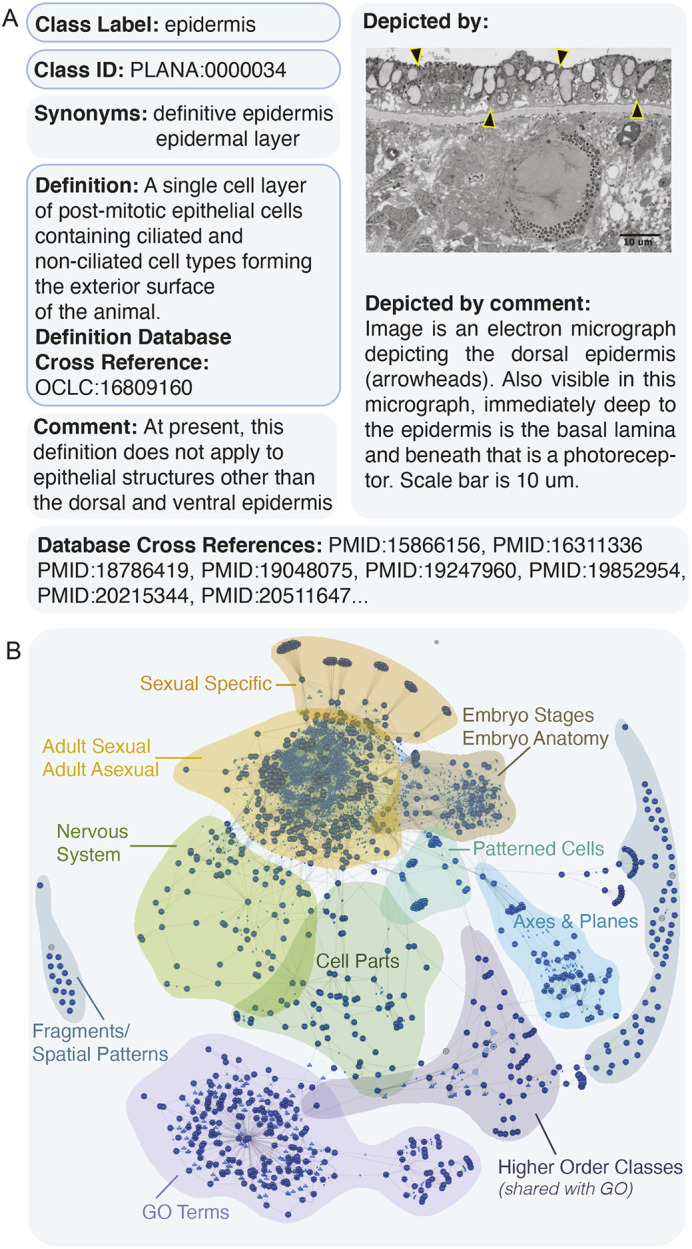
**PLANA class annotation fields and structure.** (A) An example of required (blue outlines) and optional annotations for the class ‘epidermis’. (B) WebVOWL visualization of PLANA structure. Each class is represented by a dark blue dot. The proximity between classes is a metric of similarity and relationships between classes (object property-based axioms). Clusters of classes are noted with their categories.

Each class was annotated with required information as follows: a singular name (label), e.g. ‘eye’, a unique ID, a definition, and the relevant reference(s) for the definition and the relationships for each class (def_debxref; Fig. 2A). Optional annotations were also assigned to classes (Table 1), including synonyms, external ontology database identification numbers (dbxref) to facilitate comparative anatomy searches, and images depicting anatomical features, along with explanatory legends and references (Fig. 2A). For each annotation field, multiple entries are permitted. Together, all classes and their relationships comprise a large, self-organizing webwork (Fig. 2B).

**Table 1.**
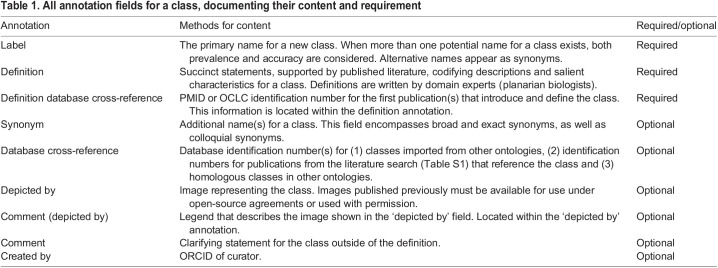
All annotation fields for a class, documenting their content and requirement

In order to extend the use of PLANA and promote interoperability with other ontologies, high-level classes from the Uberon anatomy ontology hierarchy (Mungall et al., 2012) were imported directly into PLANA (e.g. ‘anatomical entity’ UBERON:0001062 and ‘life cycle stage’ UBERON:0000105). These wide-ranging, inclusive classes confer the basic structure and frequently appear as nodes in the PLANA hierarchy (Fig. 2B). Imported Uberon classes retained all annotation fields as they appear in their origin ontology and no additional annotations were added. Importantly, these imported classes are subject to change when Uberon is updated, thus it is important they are sufficiently broad to encompass planarian anatomy accurately.

To facilitate comparative anatomy queries across species, additional classes from extant anatomy ontologies were instantiated into PLANA whenever possible, including the Common Anatomy Reference Ontology (CARO) (Haendel et al., 2008), the Biological Spatial Ontology (BSPO) (Dahdul et al., 2014), the Cell Ontology (CL) (Diehl et al., 2016), the Gene Ontology (GO) (Ashburner et al., 2000; The Gene Ontology Consortium, 2019) and Uberon (Mungall et al., 2012). Instantiation imports a class and allows modifications and additions to class annotations to reflect planarian-specific anatomical information accurately. Importantly, annotations associated with instantiated classes do not change when their ontology of origin is updated. For example, the class ‘eye’ UBERON:0000970 was instantiated into PLANA to annotate it with information about cellular origin, development and anatomical location in planarians. Instantiated terms were assigned a new PLANA ID (e.g. ‘eye’ PLANA:0000036), and the original external ontology identification number was retained in the database cross-reference (dbxref) annotation field so analogous terms remain associated. Class instantiation promotes interoperability of ontologies by ensuring analogous terms are findable (indexable), associated, and yet remain tailored to planarian anatomy.

#### Synonym annotation

Ontology interoperability and applications involving human input require PLANA to allow variability in language. This variability is accommodated by annotating classes with synonyms. Synonyms make PLANA more flexible for users, enabling queries and searches to be more inclusive. When synonymous names were present in the publication record, class labels were assigned to the most commonly used term, and less frequently used names were annotated as synonyms. For example, ‘cephalic ganglia’ PLANA:0000044 has the synonyms brain, cerebral ganglia, and bi-lobed brain. Classes may be annotated with multiple synonyms (Fig. 2A) and synonyms for this release were only taken from the literature search (Table S1). Exceptions to this rule include instances in which classes were imported from another anatomy ontology and represented a broad comparative anatomical name. For example, ‘eye’ PLANA:0000036 (UBERON:0000970) superseded the popular moniker photoreceptor as the class name in PLANA in order to strengthen cross-species comparisons. Popular names that lacked specificity were not used as class labels, e.g. ‘epidermis’ (the outermost epithelial covering of the animal) was selected as a class name rather than the often-used term epithelium, as there are many other epithelial tissues apart from the ‘epidermis’.

#### Class definitions and reference annotations

In order to clarify both class structures and meaning for planarian biologists, comparative anatomists, and ontologists alike, each class has a written definition embedded with corresponding published reference(s) demonstrating the first use of the term in our literature search (def_dbxref, Fig. 2A, Table S1). Original external ontology ID(s) from instantiated classes are held in a separate database cross-reference (dbxref) annotation field. The dbxref field also contains PubMed identification numbers for articles from the literature search that contain that class (Fig. 2A, Table S1). Taken together, the class definitions and database cross-references provide provenance and promote ontology interoperability*.*

#### Prototypic imagery

Through its definitions and links to publications, PLANA is inherently an educational resource. To increase PLANA's didactic potential, we appended images to classes using the optional ‘depiction’ annotation field and added explanatory legends using the ‘comment’ annotation field (Fig. 2A). Over 200 classes in this release are accompanied by an archetypal image, either an illustration for spatial classes or an electron or light microscopy image for anatomical structures. This imagery augments the written class definition through clear visualization (Fig. 2A).

#### Composite classes

**Fig. 3.**
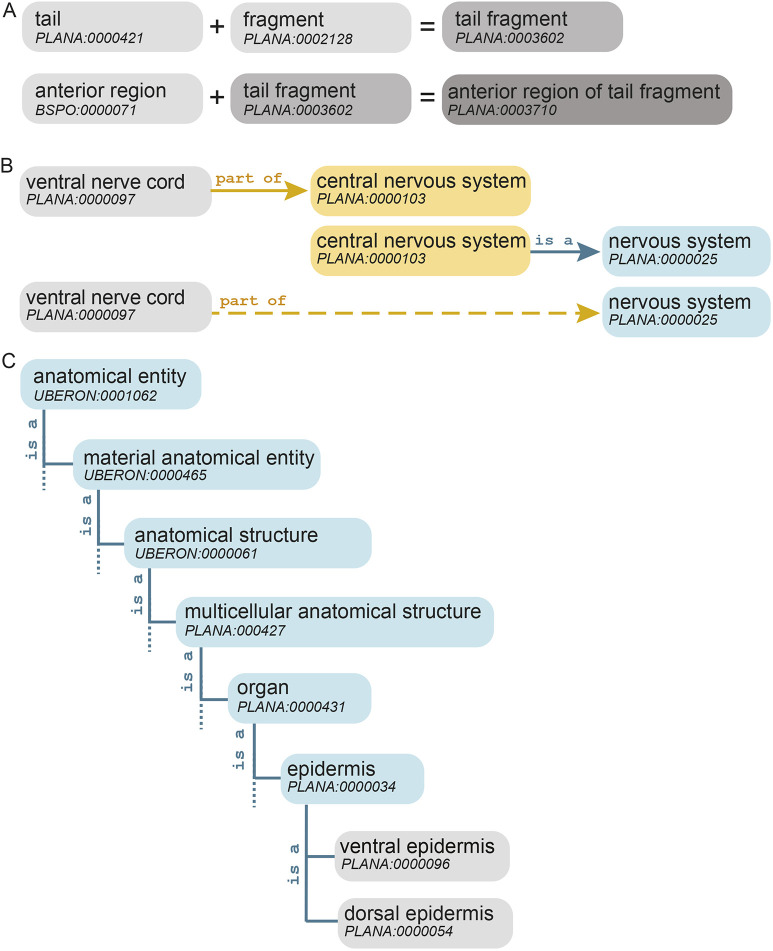
**Creation of new classes using patterning algorithms and relationship.** (A) Composite classes, e.g. ‘tail fragment’ PLANA:0003602, generated by patterning algorithms, may be used to create new classes with greater specificity, e.g. ‘anterior region of tail fragment’ PLANA:0003710. (B) Object property transitivity allows relationships to be inferred indirectly, across multiple layers of the PLANA hierarchy. Solid arrows are asserted axioms in PLANA, whereas the dashed arrow is an inferred relationship. (C) Relationship hierarchy for the dorsal epidermis and ventral epidermis classes through the ‘is a’ relationship.

During the literature survey to generate class names (Table S1), multi-word classes were included (e.g. ‘photoreceptor neuron’). However, the need to create multiple terms that follow a common pattern became apparent (e.g. ‘anterior photoreceptor neuron’ and ‘posterior photoreceptor neuron’). It became clear that many classes could be created as needed using an additive, formulaic approach already employed by other ontologies. Pre-composed or composite classes (Mungall et al., 2010) were created using patterns that auto-generate a new class by combining two existing classes using Dead Simple OWL Design Patterns (DOSDP) (Osumi-Sutherland et al., 2017) (Fig. 3A). Composite definitions were auto-generated and may be overwritten by curators. Furthermore, composite classes may be used to generate new classes, providing a stereotypical way to generate terms with greater specificity (Fig. 3A). Patterns used to make composite classes appear in Table S3. This automated addition of classes allows rapid expansion of more specific classes as the need for spatial and temporal granularity grows.

### Constructing a relational structure: relations and object properties

An ontology's strength lies within its hierarchical structure, which is provided by a single baseline categorical relationship (‘is a’) (Fig. 3C) working together with more specific relational terms called object properties. This release of PLANA uses 14 object properties, all from the Relationship Ontology (RO) (Smith et al., 2005), which enable the construction of categorical, spatial, developmental and temporal relationships between classes (Table 2, Materials and Methods). Following convention, object properties are herein formatted using lowercase lettering and the font Courier New (Van Slyke et al., 2014). For example, the ‘ventral nerve cord’ is part of the ‘central nervous system’. Some object properties have the feature of being transitive, meaning the property can be inherited by a subclass, or entailed through the hierarchy. To expand on the previous example, part of is transitive; because the ‘central nervous system’ is a ‘nervous system’ and the ‘ventral nerve cord’ has been asserted as being part of the ‘central nervous system’, ‘ventral nerve cord’ can also be inferred to be part of the ‘nervous system’ (Fig. 3B). To maximize inference from minimal information, we assigned transitive relationships spanning one level of anatomical organization.

**Table 2.**
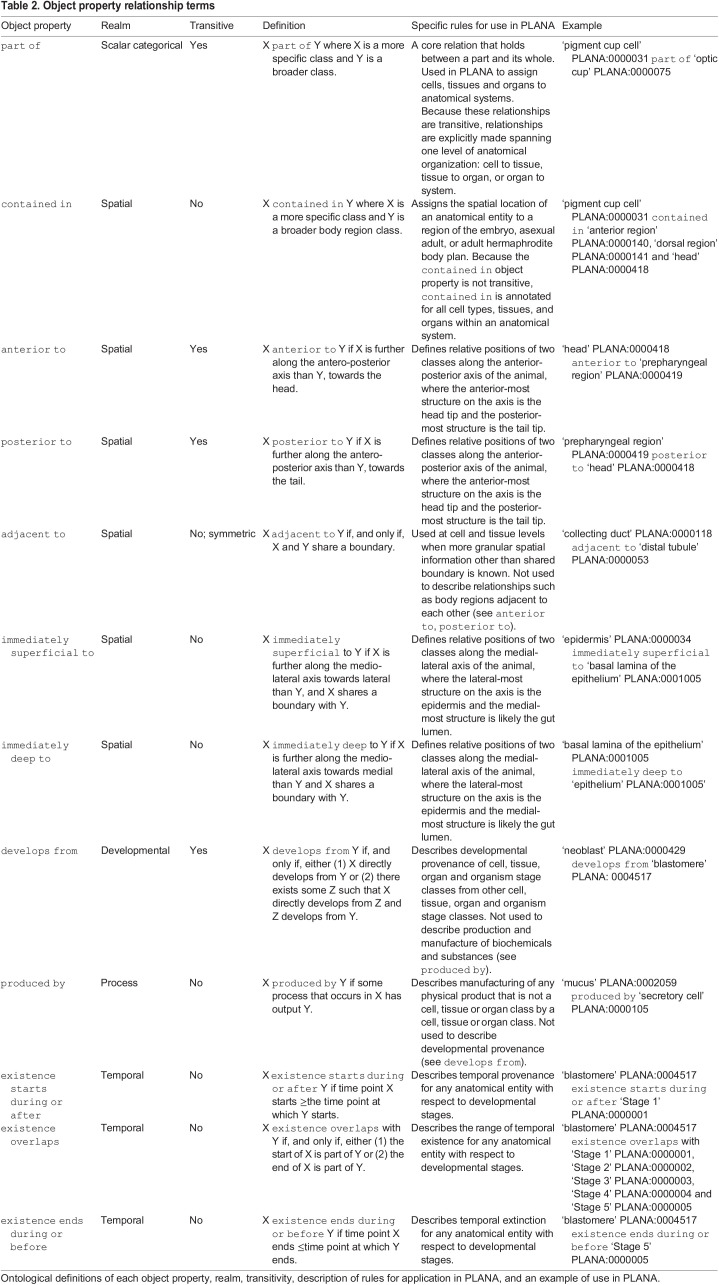
Object property relationship terms

**Fig. 4.**
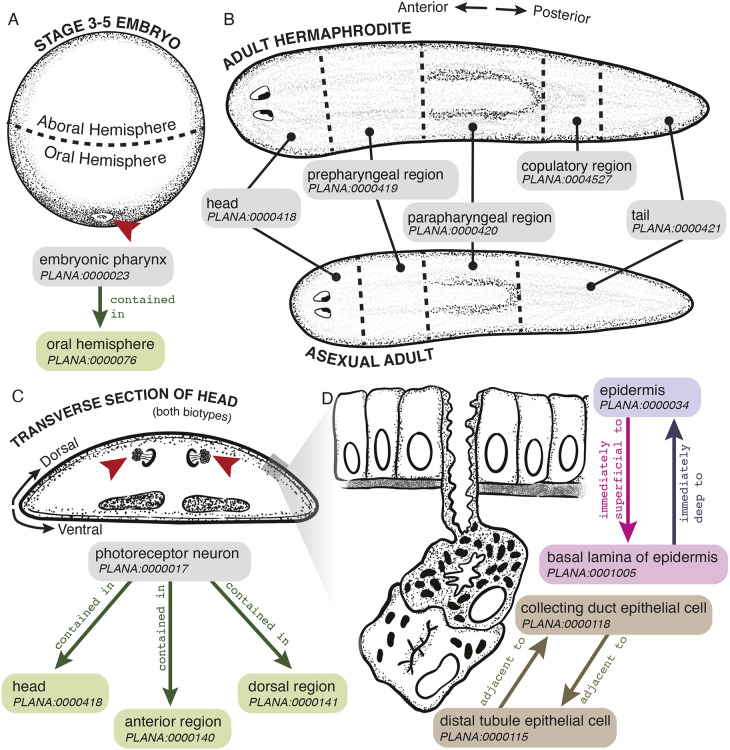
**Codifying spatial relationships using the contained in object property.** (A,B) *Smed* embryonic (A) and adult (B) body plans. (A) The ‘embryonic pharynx’ (red arrowhead) is contained in the ‘oral hemisphere’ of ‘Stage 3’, ‘Stage 4’ and ‘Stage 5’ *Smed* embryos. (B) Body region classes for the ‘adult hermaphrodite’ and ‘asexual adult’. (C) ‘Photoreceptor neuron’ (red arrowheads) is contained in the ‘anterior region’, the ‘dorsal region’ and the ‘head’. Gray box denotes epidermal and subepidermal region depicted in D where the ‘epidermis’ is immediately superficial to the ‘basal lamina of the epidermis’, which is in turn immediately deep to the ‘epidermis’. Another spatial relationship is that the ‘collecting duct epithelial cell’ and ‘distal tubule epithelial cell’ are adjacent to each other.

**Fig. 5.**
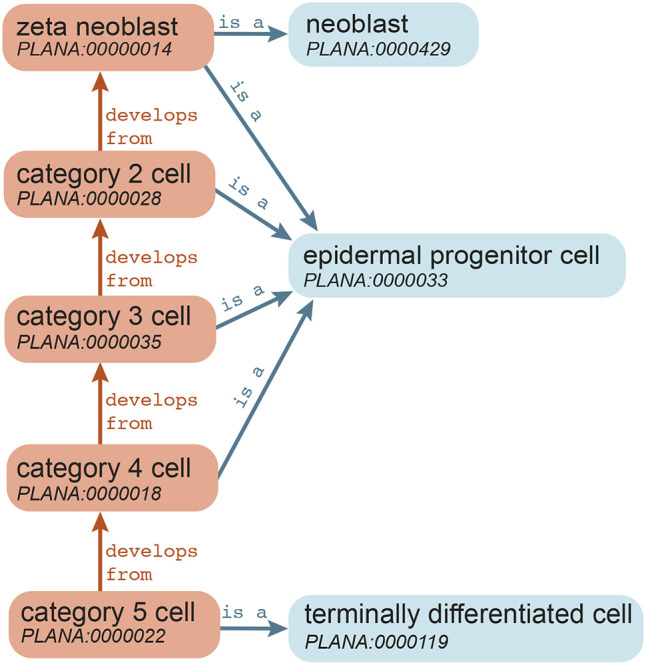
**Ontogeny is recorded using the develops from object property.** Schematic showing both the is a relationship and develops from object property charting a proposed lineage trajectory for the epidermal lineage, from stem cell to terminally differentiated cell type.

Object properties in PLANA codify categorical, spatial, developmental and temporal relationships between classes. Spatial information that associates cell, tissue, organ and anatomical system classes with defined regions of the intact embryo, juvenile or adult body plans is represented by the contained in object property (Fig. 4A,B). Examples include ‘embryonic pharynx’ contained in ‘oral hemisphere’ (Fig. 4A), and ‘photoreceptor neuron’ contained in ‘anterior region’, ‘dorsal region’ and ‘head’ (Fig. 4C). Adjacent spatial information is set using the reciprocal immediately superficial to and immediately deep to object properties (Fig. 4D). Developmental relationships and lineage trajectories are set by the develops from object property. A well-studied example, the epidermal lineage, is documented as follows: ‘Category 2 cell’ develops from ‘zeta neoblast’, ‘Category 3 cell’ develops from ‘Category 2 cell’, ‘Category 4 cell’ develops from ‘Category 3 cell’, and ‘Category 5 cell’ develops from ‘Category 4 cell’ (Fig. 5) (Eisenhoffer et al., 2008; Pearson and Sánchez Alvarado, 2010; van Wolfswinkel et al., 2014; Tu et al., 2015; Cheng et al., 2018). ‘Zeta neoblast’ and Category 2, 3 and 4 cells are all an ‘epidermal progenitor cell’, whereas Category 5 cells are a ‘terminally differentiated cell’ as set by the hierarchical categorical ‘is a’ relationship.

### Checking the structure: benchmarking and queries

To assess PLANA's structure objectively, both internally and externally, we ran iterative queries within PLANA and compared PLANA to extant anatomy ontologies with benchmarking metrics. To internally assess and ensure the veracity of asserted and inferred axioms, we systematically queried PLANA using the competency questions listed in Table S4. When a query return contained errors, such as incorrect relationships between classes (e.g. when ‘ovary nerve plexus’ part of ‘asexual adult’ was returned), asserted axioms were edited and/or added to correct the inferred error. This process was iterated until all returned classes were correct.

To ensure comparable coverage of PLANA with respect to other anatomy ontologies, we checked multiple metrics for benchmarking. Like other anatomy ontologies, PLANA's broadest class starts at the organism level (‘whole organism’ PLANA:0000136), and its most granular classes are subcellular components (e.g. organelles; Fig. S1A). Comparison of class number between anatomy ontologies reflects differences in (1) gross anatomy between species, (2) depth of current anatomical research and (3) field-specific use (i.e. neuronal mapping in *Drosophila*). As expected, given these caveats, PLANA (863) contains more classes than the Ctenophore (646), Tick (629), Echinoderm (497) and *Dictyostelium* (134) anatomy ontologies, and less than Mouse (adult: 3257, development: 8643), *Xenopus* (1763), Zebrafish (3219) and *Drosophila* (17484) (Fig. S1A). Notably, 61% of *Drosophila* anatomy ontology classes are nervous system components, a feature that reflects its usage to curate high-resolution maps of the fly nervous system (Fig. S1A). Despite PLANA's smaller class count, its relative complexity and deployment of relationships (axioms/class) is, in fact, greater than the heavily used Uberon, CL, and *Drosophila* ontologies (Fig. S1C). Additionally, the relative ratio of synonyms to classes is equivalent to other ontologies (0.72 average synonyms per class; Fig. S2A). A third of PLANA (34.2%) class labels and synonyms corresponds to those of highly used anatomy ontologies (Fig. S2B).

Taken together with our effort to comprehensively identify used classes and synonyms in published works (Table S1), these benchmarking and coverage metrics suggest PLANA's class counts are on a par with other anatomy ontologies (Fig. S1B). Moreover, PLANA outperforms all anatomy ontologies sampled with respect to the sheer amount of information codified using relationship terms (average axioms per class; Fig. S1C).

### Talking to other ontologies: interoperability

Bespoke, field-specific ontological frameworks are useful for data organization, but become extensible and more powerful when designed to work with other ontologies. Optimal interoperability between PLANA and other ontologies was achieved by importing higher-order parent classes from Uberon and instantiating classes from other ontologies, while recording their original ID as a dbxref annotation. Interoperability is also provided by including ‘homologous’ classes from other anatomy ontologies in the dbxref annotation, for example ‘basal lamina of epithelium has 15 ‘homologous’ classes (Table 1; Fig. S3A). At present, 230 PLANA classes have a total of 362 dbxrefs from 56 other ontologies, the majority of which are from GO (21.3%), Uberon (19.1%) and CL (14.1%) (Fig. S3B).

Interoperability is also built into composite classes because BSPO (Dahdul et al., 2014), GO (Ashburner et al., 2000; The Gene Ontology Consortium, 2019) and Phenotype and Trait Ontology (PATO) (http://www.obofoundry.org/ontology/pato.html) classes were imported into PLANA upon creation of composite classes. For example, GO terms for mitotic and meiotic cell cycle phases were imported to generate PLANA composite classes for stages of the neoblast cell cycle (e.g. ‘S phase neoblast’), the mitotic germ cell cycles (e.g. ‘metaphase spermatogonium’) and meiotic germ cell cycles (e.g. ‘meiotic metaphase 1 stage spermatocyte’) (Table S3). At present, 160 GO terms are used in PLANA and they largely occupy organelle level classes and cell cycle phases (Table S5).

Although direct import and instantiated use of classes from other ontologies is important for interoperability, another equally fundamental means of ensuring that one ontology can talk to another is through limiting object properties to those referenced in the Relation Ontology (RO) (Smith et al., 2005). The RO is a reference set of relations and their semantics used for standardization across ontologies in the Open Biological and Biomedical Ontology (OBO) Foundry (Smith et al., 2007). Our strict use of RO object properties ensures that PLANA relationships are found in, and stated similarly to, other ontologies. PLANA was constructed with an eye towards ontology interoperability, facilitating its application to evo-devo and comparative anatomy studies. Interoperability will also promote future extension and application of PLANA as a base framework for multiple types of data organization and will allow other ontology builds to use PLANA efficiently.

### PLANA in action: organization of gene expression data

**Fig. 6.**
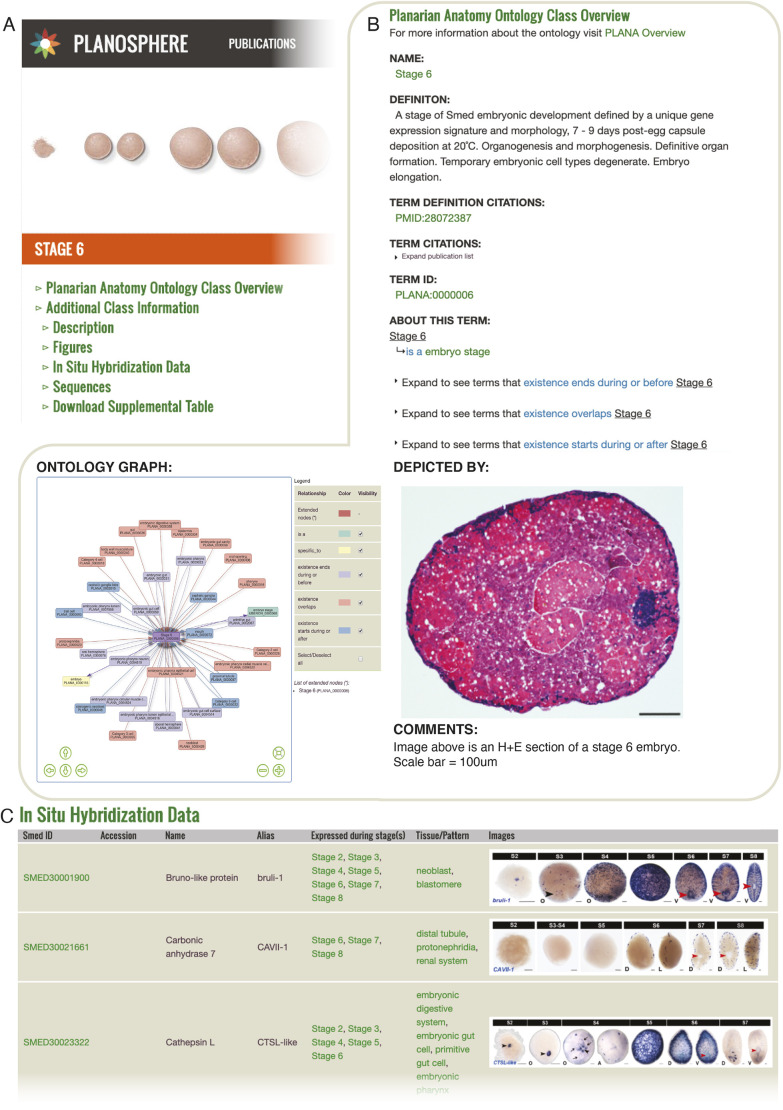
**PLANA was used to create web-based resources for *Smed* embryogenesis.** (A) Overview of didactic tools for *Smed* embryogenesis that rely upon PLANA for organization and presentation of the data. (B) Staging Resource overview. Webpage for ‘Stage 6’ PLANA:0000006 displays PLANA metadata and the Ontology Graph visualization tool. (C) Whole-mount *in situ* hybridization data were annotated and organized using PLANA classes.

As publications generate large amounts of data, there is an increasing need to make this data available and searchable in centralized locations. Planosphere is an online resource aggregator for published *Smed* datasets generated by members of the Sánchez Alvarado lab. We demonstrated PLANA's utility for organizing and mining large datasets by applying PLANA to the organization of an embryonic staging series and a molecular fate-mapping atlas on Planosphere. Each PLANA class has its own web page on Planosphere, ensuring seamless integration of the PLANA hierarchy and class metadata into these resources (Fig. 6A).

#### Educational resources for planarian embryogenesis

Planosphere hosts two tools powered by PLANA for exploring *Smed* embryogenesis: a staging series (https://planosphere.stowers.org/staging) and a molecular fate-mapping atlas (https://planosphere.stowers.org/atlas) (Davies et al., 2017). The staging series defines and describes the eight stages of *Smed* embryogenesis, incorporating single embryo RNA-Seq gene expression data along with chronological and morphological information. The molecular fate-mapping atlas documents cell and tissue types unique to early embryonic stages, as well as the development of adult anatomical systems. Published gene expression data from the single embryo RNA-Seq developmental time course and whole-mount *in situ* hybridization experiments on staged, wild-type embryos were annotated using PLANA. For the staging series, transcripts with enriched expression at each stage were annotated with relevant PLANA class(es) (Stages 2-8). For the fate-mapping atlas, PLANA classes for the biotype, life cycle stage(s) and anatomical structure(s) positive for expression were linked to transcripts (Fig. 6B). Use of PLANA to curate gene expression data enables users to search by primary sequence, transcript identifier/name, developmental stage and anatomical site(s) of expression, from cell type to anatomical system. Hyperlinks facilitate rapid navigation to transcript webpages (Transcript Pages) and PLANA class webpages (Fig. 6C), enabling users to hone or broaden their queries, and to access relevant background information concerning embryonic anatomy and development.

#### Planarian anatomy gene expression (PAGE)

**Fig. 7.**
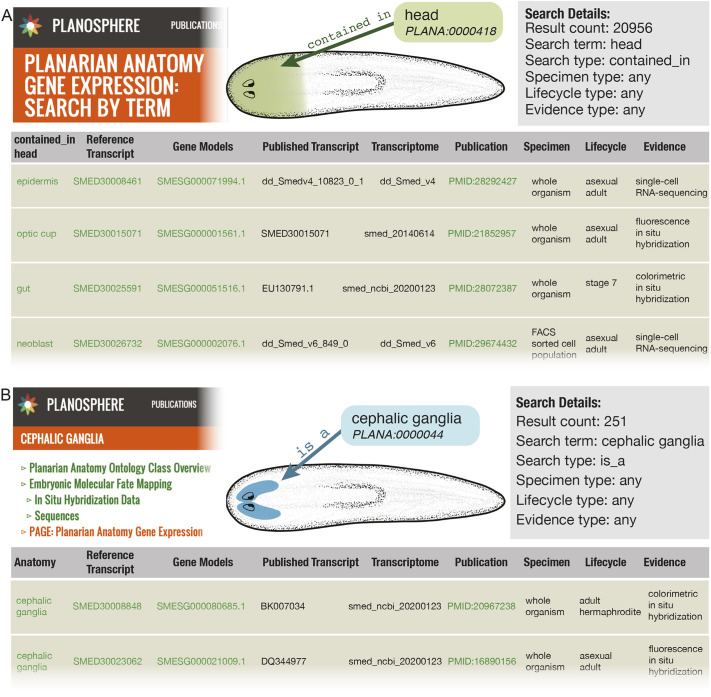
**Planarian Anatomy Gene Expression database.** (A) The PAGE resource is accessible via the Planosphere website and returns a downloadable table for searches such as: find all transcripts annotated as expressed in anatomical structures contained in the head across all lifecycle stages, specimen type and evidence types. Search by transcript or publication not shown. (B) PAGE is incorporated into individual class webpages under the PAGE: Planarian Anatomy Gene Expression section. For example, the Cephalic Ganglia web page includes references, genes and transcripts that are annotated as being expressed in an anatomical structure that is a cephalic ganglia.

We used PLANA to create the Planarian Anatomy Gene Expression (PAGE) database, a web-based resource that allows users to mine published gene expression data using ontological inference and PLANA classes (https://planosphere.stowers.org/search/page/about; Fig. 7A). Our PAGE web forms enable users to do complex searches by term, transcript or publication that would traditionally involve extensive literature research and elaborate manual documentation. Tasks such as identifying all transcripts expressed, across transcriptomes and research laboratories, in any structure that is part of the ‘central nervous system’, or all structures a single transcript or a group of transcripts have been published as being expressed in, now takes seconds.

To generate the PAGE database, we curated qualitative expression data from 155 publications cited in the literature review (Table S1) to collect the following information: gene name(s), transcript identification number(s), GenBank accession number(s), PubMed identification number for the citation, evidence classes from the Evidence and Conclusion Ontology (ECO) (Chibucos et al., 2017; Giglio et al., 2019) (e.g. ‘colorimetric *in situ* hybridization evidence’, ‘fluorescence *in situ* hybridization evidence’, ‘RNA-sequencing evidence’, ‘single-cell RNA-sequencing evidence’ and ‘cDNA to DNA expression microarray’), PLANA class(es) describing anatomical site(s) of expression, and curator ORCID. In total, 74,853 instances of expression from wild-type, intact animals and sorted cell populations were manually curated in the PAGE database. Expression data in regenerating body fragments and in RNAi knockdown animals were not collected.

Because accessions and identifiers for annotations came from multiple transcriptomes and other sources, such as expressed sequence tags, we built a translation tool, Rosetta Stone Transcript Mapper, to map all sequences back to the smed_20140614 transcriptome (https://planosphere.stowers.org/search/rosettastone/blaze; Fig. S4) (Tu et al., 2015). Although any transcriptome could have been selected as a reference, the smed_20140614 transcriptome was used because it is currently the transcriptome of choice for next-generation sequencing mapping in our laboratory. PLANA itself is reference independent and can be used in conjunction with any transcriptome. Using Rosetta Stone Transcript Mapper, the 74,853 annotations referenced 30,715 unique accessions. Those accessions mapped to 16,657 transcripts in the reference transcriptome, which are associated with 15,513 gene models (Grohme et al., 2018). PAGE is searchable by any anatomical term in PLANA (including synonyms), by transcript or accession number, and by publication. PAGE covers 19.6% (168) of PLANA anatomical terms that largely encompass body region, cell and tissue level terms (Table S6).

Using the PAGE resource, one with a broad interest in transcripts annotated with a PLANA class that is contained in the ‘head region’ would retrieve a downloadable list of 20,956 instances of expression data from 123 different publications spanning seven life cycle stages; five types of evidence; 44 PLANA classes; 15 different published transcriptomes; and 8944 unique reference transcripts, associated with 7473 gene models (Fig. 7B). Alternatively, one with a narrow interest in a specific transcript can search PAGE by transcript, e.g. ‘dd_Smed_v6_76069_0_1’. This search returns a set of six transcript IDs from four different transcriptomes and seven publications. All of the transcripts are described as *ovo* and documented by three evidence types as being expressed in six anatomical structures in a sliding scale of specificity from ‘photoreceptor neuron’ to ‘eye cell’ to ‘head region’ (Table S7); all of these classes are part of ‘eye’ and thus contained in ‘head region’.

Aside from providing individual records of expression, PAGE provides an initial platform to survey the expression landscape. As expected, the majority of the expression information in PAGE was obtained using high-throughput methods: single-cell RNA-Seq (48.3%, 20,089 entries) and RNA-Seq (34.1%, 14,185 entries). Colorimetric and fluorescent *in situ* hybridization and cDNA microarrays make up the remainder of PAGE database entries with 9.1% (3774 entries), 1.4% (570 entries) and 7.1% (2929 entries), respectively. PAGE has at least one instance of evidence recorded for 49.6% of the smed_20140614 transcriptome and 67.1% of the smesg gene models. GO enrichment of transcripts recorded in PAGE returned 8032 terms and revealed that the top three GO terms by adjusted *P*-value were: ‘cell differentiation’, ‘cellular developmental process’ and ‘animal organ development’ (Table 3; Tables S8, S9). GO enrichment of transcripts not included in PAGE cover only 364 GO terms with the top three by corrected *P*-value being ‘DNA integration’, ‘transposition’ and ‘catalytic activity’, acting on DNA (Table 3; Tables S10, S11).

**Table 3.**
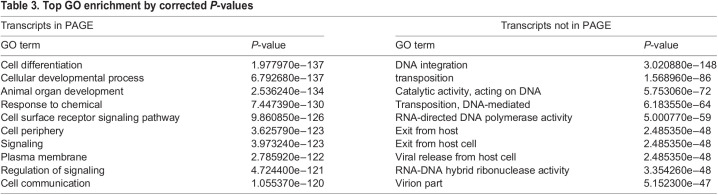
Top GO enrichment by corrected *P*-values

### PLANA in the future: new contributions and versions

PLANA is a living resource. Changes to PLANA will be made by manuscript authors and stakeholders in the community to reflect advances reported in future publications. New releases are automatically scheduled for weekly pick-up by Open Biological and the Ontology Lookup Service (OLS) (Table S5). PLANA will be versioned following substantive changes to the structure or monthly to pick up small changes. Queries (Table S4) will be performed for quality control prior to the release of each new version.

Members of the research community are encouraged to assist with PLANA curation through submission of a new class(es) and/or proposing edits to annotations for existing class(es), such as synonyms and PMIDs, using the GitHub issue tracker (https://github.com/obophenotype/planaria-ontology/issues). New class submissions require a class name, definition, PMID or DOI numbers for publication(s) referencing the definition, and a contact name and email address for the contributor. Two curators will review new classes and other proposed edits and will correspond with the contributor to resolve outstanding questions prior to updating PLANA. Bulk requests for new classes should be submitted using the spreadsheet template posted on the PLANA GitHub issue tracker. Requests without published evidence upon submission will not be accepted.

When issues consist of changes that are unanimously agreed to by the reviewers and have a citation for supporting evidence, the changes and/or insertions will be made after a minimum of a 1 week waiting period. This waiting period is used to monitor discussions for and against the proposed changes in the GitHub issue forum. If no discussion is created on a pending issue, the changes will be made as per the request. When proposed changes, with published evidence, generate contradictory discussions within the 1 week waiting period, the arguments will be evaluated by a third researcher in the field (requested via email) and a decision by the new reviewer will be made, with discussion and reasoning posted on the Issue Tracker page. If the reviewers cannot agree, and a long-term solution cannot be agreed upon, the issue will be addressed in a session at the next International Planarian Meeting. Following and participating in the discussions on proposed changes to PLANA is open to all, and only requires users to sign up for a free GitHub account and ‘watch’ the PLANA repository. Watchers will receive email notifications when issues and discussions arise and when PLANA is updated.

The PLANA GitHub repository issues page contains a searchable history of questions and resolutions to issues raised by curators and community members. Questions may be submitted by opening a new issue to ensure the discussion and decision-making process is open, transparent and archived. Requests to deprecate class(es) should be made by opening an issue. Obsolete classes remain visible in future versions of PLANA as deprecated classes. When a class is superseded by a new class, the deprecated class is listed as a synonym for the new class.

### Accessing and editing PLANA

The latest version of PLANA can be downloaded through the OBO Foundry (http://www.obofoundry.org/ontology/plana.html) or the GitHub repository (https://github.com/obophenotype/planaria-ontology) and all edits to PLANA are made in the GitHub repository through the issue tracker.

### Viewing PLANA

PLANA can be browsed on Planosphere (planosphere.stowers.org/anatomyontology). The PLANA class glossary can be searched and is linked to class webpages (https://planosphere.stowers.org/ontology). Each class webpage contains the PLANA ID, definition and citation(s), tools for visualizing annotated object property relationships, and tables with planarian transcripts known to be expressed in each class (see below). The European Bioinformatics Institute (EMBL-EBI) Ontology Lookup Service (OLS) tree (https://www.ebi.ac.uk/ols/ontologies/plana) depicts hierarchical relationships among PLANA classes. An interactive feature, Ontology Graph, dynamically depicts user-selected relationship(s) for the class of interest in either cluster or hierarchical format and generates graphic files for download (Perez-Riverol et al., 2017).

WebVOWL (visualdataweb.de/webvowl/#iri=http://purl.obolibrary.org/obo/plana.owl), an interactive ontology visualization tool, may also be used for exploration and graphical depictions of PLANA.

### Reporting

PLANA is described according to the Minimum Information for the Reporting of an Ontology Guidelines (Matentzoglu et al., 2018) (Table S12). PLANA is supported by the Sánchez Alvarado Lab at the Stowers Institute for Medical Research in Kansas City, Missouri, and data are licensed under a Creative Commons BY-NC 2.0 License. When using PLANA, the date(s) and/or version number(s) for the relevant PLANA files should be reported.

## Conclusions

The planarian research community is generating transcriptomic, genomic and phenotypic data at a rate that is already well past the limited amount of raw material human brains can hold, let alone infer information from. Although databases can tackle the problem of information quantity, databases cannot infer attributes based upon known relationships. To mimic what the human brain does so well (quickly infer relationships among categories that are made by binning according to properties), we created an ontology framework to organize and facilitate inferential searching of anatomy-related data. PLANA addresses two critical needs in our field: (1) PLANA provides a controlled anatomical vocabulary based on current field use; (2) PLANA is an open-source, adaptable, extensible framework module that researchers can use to create new tools for universal and standardized data organization and aggregation, thus promoting searchability within and among large data sets.

We showcase the power and versatility of PLANA to organize data by using it to structure extant *Smed* gene expression data in the *Smed* embryogenesis molecular staging series and fate-mapping atlas, as well as the PAGE community expression tool.

The PAGE database associates PLANA classes with an integrated reference for *Smed* transcripts and gene models that readily allows users to assess equivalency and make connections for spatial expression patterns and digital gene expression data produced across different platforms. Using PAGE, one can quickly ascertain whether whole-mount *in situ* hybridization data has been reported for single-cell RNA-Seq cluster-enriched biomarkers. In addition to PAGE, PLANA has been used to annotate personally curated, image-based expression patterns in PlanGexQ (Roy et al., 2020). Furthermore, PLANA will be used to annotate high-resolution anatomical data from serial blockface scanning electron microscopy datasets.

PLANA will be instrumental to the construction of additional community resources and tools, notably the *Smed* phenotype ontology. PLANA's interoperability will facilitate the incorporation of a *Smed* phenotype ontology into uPheno and Monarch (Shefchek et al., 2020), a semantic-based integrative data platform that connects expression and phenotypes with genotypes across species. Interoperability among Uberon, Monarch, PLANA and a *Smed* phenotype ontology will facilitate comparative anatomy queries and cross-species genotypic and phenotypic comparisons.

## MATERIALS AND METHODS

### PLANA construction

PLANA content was amassed through the review of 200 publications (Table S1) to ensure comprehensive coverage of all anatomical entities reported by the planarian research community. The primary literature search was carried out in PubMed (search terms: planaria, Smed, *Schmidtea mediterranea*) and papers were not included if they were from species other than *Smed*. Papers from 2005 (advent of large screens) to 2019 and a few landmark works prior to that period were included; reviews and protocol papers were excluded. A few papers were *Smed*-specific, but contained either no anatomical terms or very few anatomical terms that were broad and redundant (e.g. ‘head’) and those papers were not included. Terms determined to be synonyms were annotated as ‘exact synonyms’ rather than ‘broad synonyms’ for clarity. All data were entered into shared Google spreadsheets. WebProtégé, because of its ease of use and Google Docs-like collaborative properties, was used with an initial draft version of the ontology to flesh out the underlying structure (Tudorache et al., 2013). Where possible, extant classes were imported from other ontologies and instantiated in PLANA.

All tools used or generated for this manuscript that have a repository or a website are cataloged in Table S13. PLANA was initialized and is maintained with the use of the Ontology Development Kit (ODK; Table S13). ODK sets up the directory and file structure and provides scripts to manage and maintain an ontology. It integrates DOSDP (Osumi-Sutherland et al., 2017) for generating terms using patterns and ROBOT (Jackson et al., 2019) (Table S13) for handling imports from other ontologies, file format conversions, and validations. DOSDP uses yaml formatted patterns (Table S3) to generate similarly structured classes, such as ‘testis cell’, ‘eye cell’, ‘pharynx neuron’ and ‘pharynx muscle cell’. These patterned terms were generated by combining two existing classes: an anatomical structure, e.g. ‘testis’, ‘eye’, ‘pharynx’, and a cell type e.g. ‘cell’, ‘neuron’ and ‘muscle cell’. Patterns may also specify that a class needs a name, definition, reference and synonym. PLANA uses yaml patterns to manage all PLANA classes, dynamically pulling data from Google spreadsheets.

Protégé was used for visual inspection of the ontology and to query the PLANA structure (Musen and Protégé Team, 2015). Queries were run using Protégé’s DL Query with the ELK 0.5.0 reasoner (Table S4) to ensure all terms are logically related and that no errant relationships were inferred after construction of our asserted hierarchy.

### Rosetta Stone Transcript Mapper

The publications entered into the PAGE database (Table S1) used several different transcriptome and gene identifiers. In order to unify this dataset, it was necessary to map the various identifiers to each other. To create this map we selected ten transcriptomes available through PlanMine (Rozanski et al., 2019), *Smed* nucleotide sequences from the NCBI (NCBI Resource Coordinators, 2016) and dd_Smed_v4 (an older version of the dd_Smed_v6 transcriptome available on PlanMine) (Table S14). Sequences from all transcriptomes were aligned with blat (-minScore=100 -minIdentity=95) (Kent, 2002) to the most recent gene model transcripts (dd_Smes_v2) and to smed_20140614 (Tu et al., 2015). Aligned sequences were assigned to the corresponding gene models. Microarray probe sequences were aligned to reference sequences with blat (-minScore=30 -minIdentity=95) (Fig. S4).

Some publications used different name formats even when using the same transcriptome. In order to address this complication, alternative transcript names were assigned (Table S15). NCBI protein accessions and names were assigned based on their corresponding NCBI nucleotide accession. UniGene identifiers from SmedGD (Robb et al., 2008) were assigned based on their correspondence to dd_Smed_v4 identifiers, which were used in their construction. miRNAs were flagged based on sequence names and correspondence to miRBase (Griffiths-Jones, 2006; Kozomara et al., 2019). Of 35,761 unique identifiers, 34,864 were mapped to the reference sequence database. We have named the database of mappings and the tools to look up various IDs the Rosetta Stone Transcript Mapper.

### PAGE construction

PAGE Annotator, a web-based application for collecting planarian class annotations was built using R and Shiny (https://sanchezalvarado.shinyapps.io/PAGE_annotator/; R package version 1.6.0., https://shiny.rstudio.com/). R package ontologyX (Greene et al., 2017) was used for traversing the ontology tree. R packages jsonlite (Ooms, 2014 preprint) and tidyverse (Wickham et al., 2019) were used for data manipulation (Table S13).

Publications (Table S1) were split among three curators to document accession numbers of transcripts and to associate expression data with PLANA anatomical structure classes. Care was taken to use anatomical terms or synonyms from the description provided in the text. Where text description was not provided or a term was not found in the ontology, the term was either added as a class or a synonym or curators picked the most relevant term present in PLANA. For example, PLANA does not contain ‘Cathepsin positive cell’ as it is currently unclear what the exact physical anatomical structure corresponding to this state is, but as these cells are located in the parenchyma, we designated all mentions of ‘Cathepsin positive cells’ as ‘parenchymal cell’ (PLANA:30003116) (Fincher et al., 2018). For all single-cell data, we relied on the decisions of the authors regarding cutoff and enrichment. Rules for curation of literature for PAGE can be found in Table S16. PAGE has an accompanying issue tracker for requested changes or additions to curations (https://github.com/planosphere/PAGE/issues; Table S13).

Annotations were reviewed, typos identified and corrected, sequence IDs manually assigned if not computationally identifiable from the manuscript text, and all sequences mapped using the Rosetta Stone Transcript Mapper (Table S13). Sequence descriptions for the reference sequences and gene models were assigned. For smed_20140614, priority was given to GenBank descriptions (Benson et al., 2005). If GenBank descriptions were not available, they were generated using Automated Assignment of Human Readable Descriptions (AHRD; Table S13). Descriptions for dd_Smes_v2 transcripts and gene models were downloaded from PlanMine (Rozanski et al., 2019) using the InterMine query builder (Kalderimis et al., 2014; Smith et al., 2012).

The annotations, mappings and sequence descriptions were organized into a triple store (WC3; https://www.w3.org/RDF//) (Table S13) and converted to turtle formatted files (ttl). The triple store was structured using Open Biomedical Association (OBAN) principles (Sarntivijai et al., 2016). The ttl files (annotations, mappings, descriptions), along with the PLANA ontology, and Evidence and Conclusion Ontology (ECO) (Chibucos et al., 2017) owl files were loaded into a blazegraph datafile (Table S13), or journal (jnl) using blazegraph-runner (Table S13). We have Blazegraph running in a Docker (Merkel, 2014) container that is web accessible to our Planosphere web server. The Docker file was based on the LYRASIS/blazegraph docker file (Table S13). Modifications were made to import our PAGE-specific jnl and to change the name of our Blazegraph instance to PAGE.

The PAGE webform searches generate SPARQL queries (SPARQL 1.1 Query Language, https://www.w3.org/TR/sparql11-update/) from the user input data. To ensure that users can only input a PLANA term, a modified version of the OLS autocomplete widget was used (Table S13). To allow SPARQL queries to incorporate the transitivity of the PLANA Ontology hierarchy and relationships using the ELK reasoner, we also run phenoscape/owlery (Table S13) through our customized docker container planosphere/owlery-plana (Table S13). Owlery is a collection of REST web services that enable querying with an OWL reasoner and a configured set of ontologies (Table S13). Through Owlery, a SPARQL query generated from our PAGE web form which asks to find all transcripts annotated as being expressed in the ‘nervous system’ (asserted) is expanded to include its transitive relation classes such as ‘central nervous system’ and ‘peripheral nervous system’ (inferred) and also generates a new SPARQL query. This second SPARQL query is then used to query the jnl housed in our Blazegraph server.

### Gene ontology (GO) enrichment

*Smed* transcripts were assigned Gene Ontology (Ashburner et al., 2000; The Gene Ontology Consortium, 2019) terms by combining the GO annotations from UniProt/SWISS-PROT best BLAST hits (param: -evalue=0.001; db date: 20170322) (UniProt Consortium, 2019)and InterProScan (version: 5.32-71.0) (Jones et al., 2014). GO enrichment was performed using TopGO (version: 2.34.0; https://bioconductor.riken.jp/packages/3.8/bioc/html/topGO.html). *P*-values were adjusted using the Benjamini–Hochberg method (Benjamini and Hochberg, 1995).

### Animals and imagery

*Smed* anatomical descriptions were based on CIW4 asexual and sexual adults (Newmark and Sánchez Alvarado, 2002). Illustrations were made using Procreate (https://procreate.art/) and Adobe Illustrator (https://www.adobe.com/products/illustrator.html). Hematoxylin and Eosin (H+E)-stained histological sections were prepared for CIW4 asexual adults (Adler et al., 2014) and embryos (Davies et al., 2017), and images were acquired on n Olympus America Slide Scanner. Many prototypical images were produced from transmission electron microscopy (TEM), scanning transmission electron microscopy (STEM) and serial block face (SBF)-SEM datasets of CIW4 asexual animals. Images were acquired on a Zeiss Merlin SEM with a STEM detector and Gatan 3View 2XP, or a Thermo Fisher Scientific/FEI Tecnai G2 Spirit BioTWIN with Gatan UltraScan 1000 CCD camera. For TEM and STEM imaging animals were prepared as described by Cheng et al. (2018). For SBF-SEM, animals were fixed as for STEM samples with *en bloc* staining steps per Tapia et al. (2012) and Hua et al. (2015) as follows: reduced osmium incubation was performed overnight at 4°C, thiocarbohydrazide incubation at 40°C for 45 min, incubation in 1% uranyl acetate overnight at 4°C then transferred to 50°C for 2 h, and lead acetate incubation for 2 h at 50°C. Animals then were dehydrated and infiltrated as for the STEM samples using either a hard formulation of Spurr's resin (EMS) or Hard Plus resin (EMS). Fiji (Schindelin et al., 2012) was used for final adjustments and bilinear downsizing to the maximum dimension of 512.

### Accession numbers

Accession numbers of transcriptomes, microarrays and additional resources used to construct the Rosetta Stone Transcript Mapper and PAGE resource can be found in Table S14. In addition, we downloaded every *Smed* sequence in the NCBI GenBank (Benson et al., 2005) nucleotide database on January 23, 2020.

## Supplementary Material

Supplementary information

Reviewer comments
