# Supplementary material for: Planarian Anatomy Ontology: a resource to connect data within and across experimental platforms
Source: Development. 2021 Aug 2;148(15):dev196097. doi: 10.1242/dev.196097 (PMC8353266; doi:10.1242/dev.196097)
Supplement: Supplementary information [file develop-148-196097-s1.pdf]

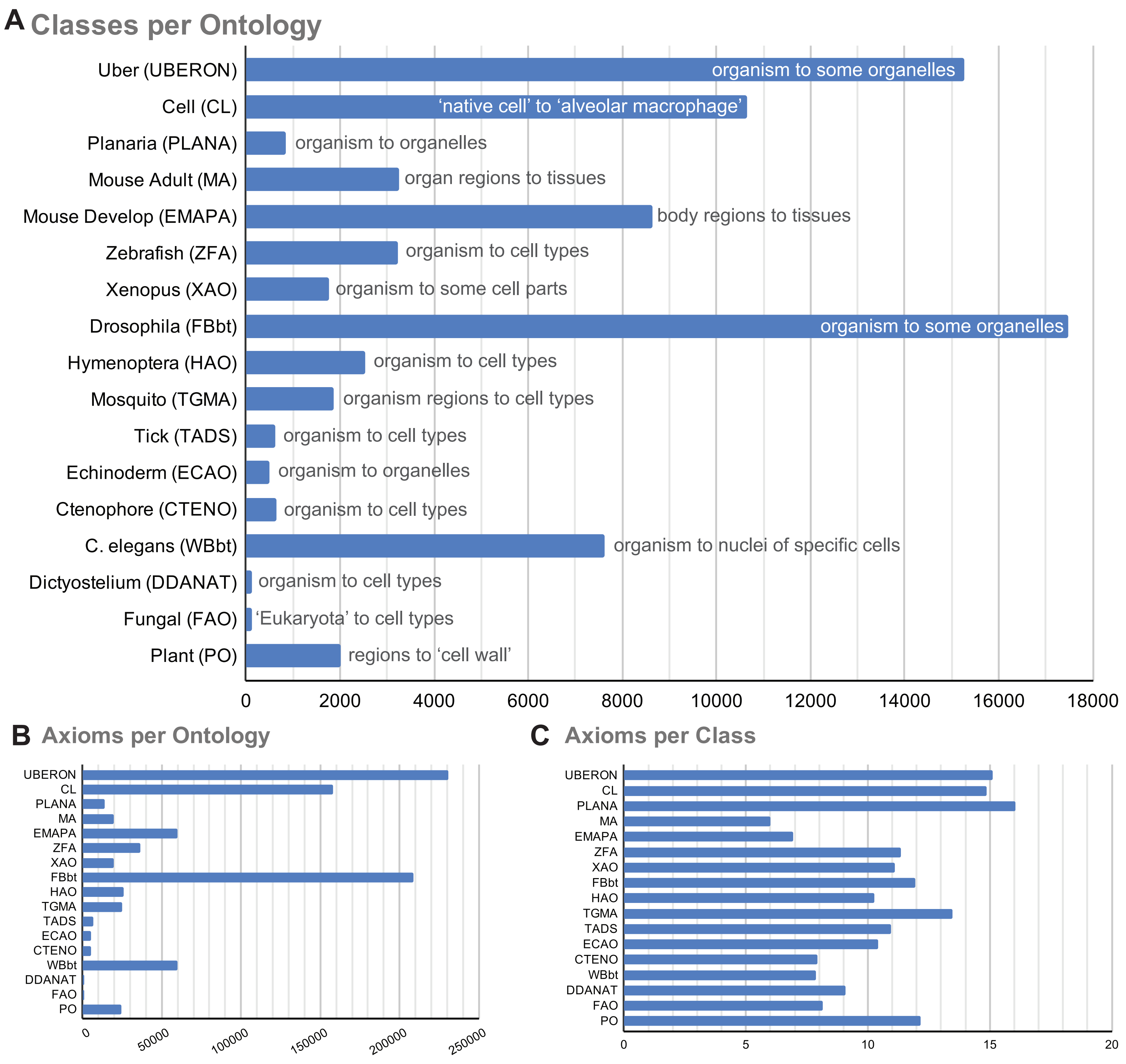

Fig. S1. Benchmark-driven comparisons of PLANA and other anatomy ontologies A)Total classes per anatomy ontology with the range of classes annotated to the right (largest scale classes/categories to smallest scale classes/categories). B) Number of axioms per ontology. C) Axioms per Class as a metric of average relational complexity. PLANA's complexity is comparable to other commonly used anatomy ontologies.

Development • Supplementary information

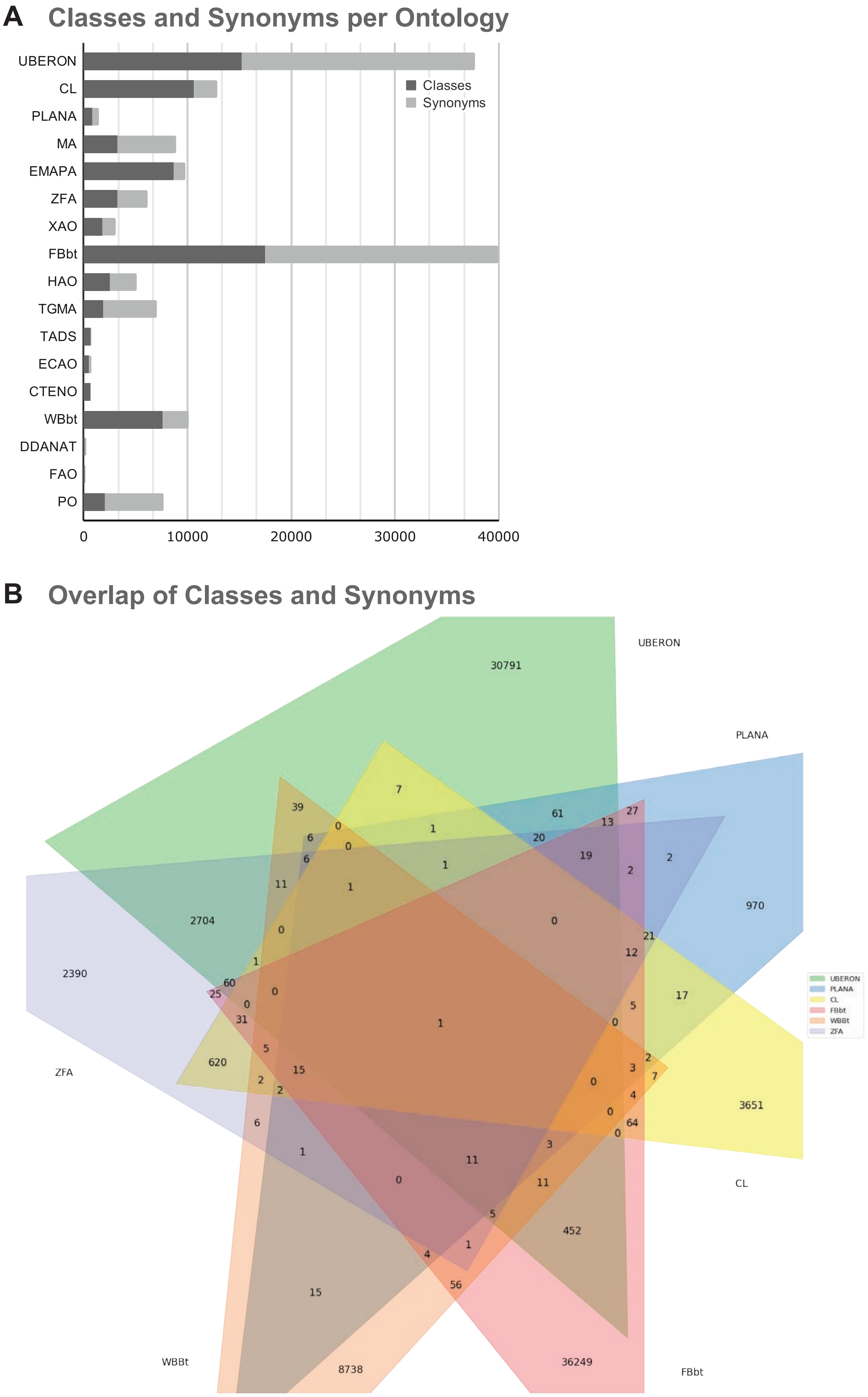

Fig. S2. Classes and synonyms between ontologies
A) Ratio of number of classes to synonyms for common anatomy ontologies. B) Overlap between ontologies by their classes and synonym.

Development • Supplementary information

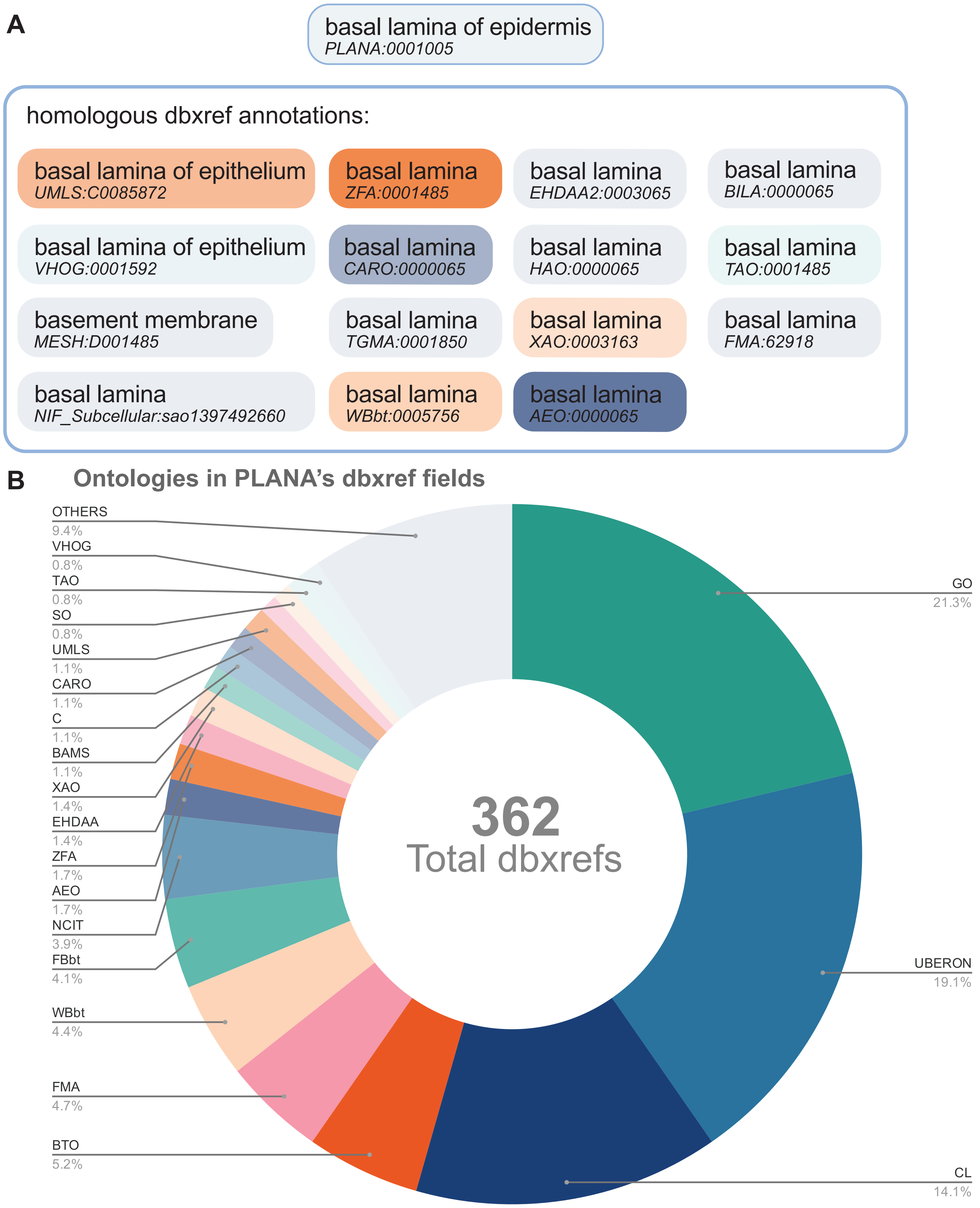

Fig. S3. Interoperability metrics

A) The dbxref annotation field confers interoperability through linkages to other anatomy ontologies, via "homologous" classes. For example, the PLANA class 'basal lamina of epidermis' has 15 "homologous" classes in 15 other ontologies. Class color reflects ontology as it is displayed in B. B) Breakdown of dbxref field by ontology referenced.

Development • Supplementary information

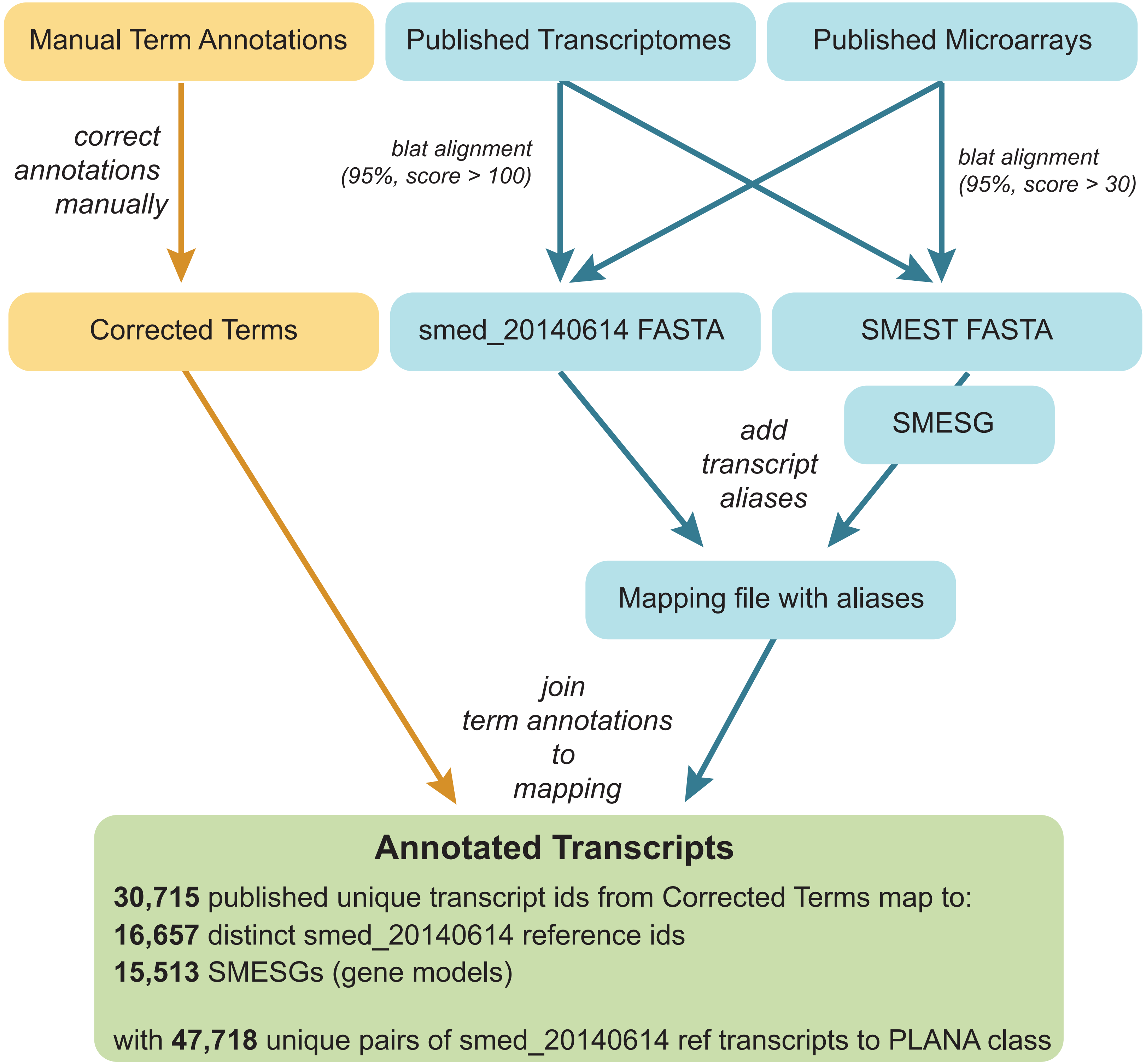

Fig. S4. Mapping workflow for PAGE
Manual term annotations were reviewed for proper accession names and fields to make a Corrected Terms set (right side, yellow). Published transcriptomes and microarrays were aligned with the reference smed_201401614 transcriptome and dd_Smes_v2 transcriptome; transcript aliases were added to create a Mapping File (left side, blue). The transcript IDs in the Corrected Terms file were then mapped to the reference transcriptome and ultimately gene models using the Mapping File.

Development • Supplementary information

Table S1. List of 200 publications comprising the literature review for Smed anatomical terms

Click here to download Table S1

Table S2. PLANA Classes and IDs used in the manuscript

Click here to download Table S2

Table S3. Composite Patterns

Click here to download Table S3

Table S4. Competency questions for DL queries

Click here to download Table S4

Table S5. GO Terms used in PLANA

Click here to download Table S5

Table S6. PAGE PLANA Term Coverage

Click here to download Table S6

Table S7. PAGE Transcript Search

Click here to download Table S7

Table S8

Click here to download Table S8

Table S9

Click here to download Table S9

Table S10

Click here to download Table S10

Table S11

Click here to download Table S11

Table S12. MIRO Reporting for PLANA

Click here to download Table S12

Table S13. All repositories referenced in manuscript

Click here to download Table S13

Table S14. Transcriptome Mapping for PAGE

Click here to download Table S14

Development • Supplementary information

Table S15. Regular Expressions used to parse transcriptome ids

Click here to download Table S15

Table S16. PAGE Curation Rules

Click here to download Table S16

Development • Supplementary information
