# Supplementary material for: Planarian Anatomy Ontology: a resource to connect data within and across experimental platforms
Source: Development. 2021 Aug 2;148(15):dev196097. doi: 10.1242/dev.196097 (PMC8353266; doi:10.1242/dev.196097)
Supplement: Reviewer comments [file develop-148-196097_review_history.pdf]

Planarian Anatomy Ontology: a Resource to connect data within and across experimental platforms

Stephanie H. Nowotarski, Erin L. Davies, Sofia M. C. Robb, Eric J. Ross, Nicolas Matentzoglu, Viraj Doddihal, Mol Mir, Melainia McClain and Alejandro Sánchez Alvarado
DOI: 10.1242/dev.196097

Editor: Kenneth Poss

Review timeline

Original submission:	18 August 2020
Editorial decision:	22 September 2020
First revision received:	22 December 2020
Editorial decision:	25 January 2021
Second revision received:	27 April 2021
Editorial decision:	27 May 2021
Third revision received:	24 June 2021
Accepted:	28 June 2021

Original submission

First decision letter

MS ID#: DEVELOP/2020/196097

MS TITLE: The Planarian Anatomy Ontology: A resource to connect data within and across experimental platforms

AUTHORS: Alejandro Sánchez Alvarado, Stephanie Nowotarski, Erin Davies, Sofia Robb, Eric Ross, Nicolas Matentzoglu, Viraj Doddihal, Mol Mir, and Melainia McClain

I have now received all of the referees' reports on the above manuscript and have reached a decision. The referees' comments are appended below, or you can access them online: please go to BenchPress and click on the 'Manuscripts with Decisions' queue in the Author Area.

As you will see, the referees express interest in your new resource, but they also have many questions and some significant criticisms. They recommend a substantial revision of your manuscript to increase the usability and value of the anatomy ontology to the community, and I agree that this is necessary before we can consider publication. If you are able to revise the manuscript along the lines suggested, which is likely to involve modification of the tools, I will be happy receive a revised version. Your revised paper will be re-reviewed by one or more of the original referees, and acceptance of your manuscript will depend on your addressing satisfactorily the reviewers' major concerns. Please also note that Development will normally permit only one round of major revision.

Please attend to all of the reviewers' comments and ensure that you clearly highlight all changes made in the revised manuscript. Please avoid using 'Tracked changes' in Word files as these are lost in PDF conversion. I should be grateful if you would also provide a point-by-point response detailing how you have dealt with the points raised by the reviewers in the 'Response to Reviewers' box. If you do not agree with any of their criticisms or suggestions, please explain clearly why this is so.

Reviewer 1

© 2021. Published by The Company of Biologists under the terms of the Creative Commons Attribution License (https://creativecommons.org/licenses/by/4.0/).

Advance summary and potential significance to field

The paper presents an ontology of planarian anatomy and development, as well as a gene expression database structured by this ontology. The source of various components in the ontology pulled from literature and existing ontologies and the process of their assembly are described in detail.
This appears to be a highly valuable resource for at least researchers within this growing field. The description of the process should also be useful to those creating novel ontologies.

Comments for the author

The presentation is clear and succinct, however given the potential interest to people building related ontologies it might help to expand details in a few places and in general further clarify the motivation for decisions, when they are noteworthy and when they are the most obvious course to at least an experienced ontology user.

Some more detail might be helpful in a couple places:

- More formally what makes something a 'top level' Uberon entry to be imported directly and subject to changes in that ontology and what makes something a lower level entry that is integrated indirectly as a copy and cross reference? I have the intuition it provides some tradeoff between stability and interoperability, but what does the particular division used achieve exactly and how is it implemented by the dividing line?

- Is there a specific strategy or rule use in applying auto composite to create entries or is this ad-hoc?

- From examples it seems like explicit use of transitive relationships is used minimally to create appropriate connected components with all nodes appropriately reachable. This seems like a logical strategy, but its not explicitly stated, is it the case? If not what are exceptions?

Couple other points of minor clarification:

- When table 2 is referenced it might increase clarity to note its contents if it does not appear on the same page, it was unclear it contained the transitivity information from the text. I presume the properties of all the relations in this table are derived directly from the Relational Ontology, if not maybe this should be clarified.
- It is worth noting if depicted by is a single entry field or if multiple illustrations of an entry are possible in theory.

<u>Reviewer 2</u>

Advance summary and potential significance to field

The manuscript by Nowotarski et al. endeavors to establish an anatomical ontology for S. mediterranea, both as a resource to the planarian research community and as an interface with other research communities. The authors align their efforts along the published ontology recommendations by Druzinsky et al. (which is cited in the text) and they make the ontology accessible via their own web resource.
Overall, this manuscript is clearly based on a multi-year effort and as stated by the authors, an anatomical ontology could indeed amount to a valuable and timely resource for the field. Moreover, the authors make provisions for community involvement, including for example a useful feedback submission procedure using the Github platform. As such, the manuscript is a suitable contribution to the "resource section" of Development. That said, concerns remain regarding the benchmarking and accessibility of the resource (see major points below). Although the authors clearly state that "…PLANA does not claim to be comprehensive or exact", they also state that they see PLANA "As a standard for anatomical information". Our major points pertain to the establishment of said standard.

Comments for the author

© 2021. Published by The Company of Biologists under the terms of the Creative Commons Attribution License (https://creativecommons.org/licenses/by/4.0/).

Major Points

A) Benchmarking

1. The authors need to compare their de novo established ontology against existing ontologies of other model systems. Metrics for the comparisons should include the number of classes and the granularity or number of child terms originating from the parent terms. This is important in order to gauge the breadth and depth of the ontology.

2. Also, it would be useful to provide the readers with an impression of the interoperability with other ontologies, i.e. the PLANA term “epidermis” is missing the synonym 'skin'. Is there a way to evaluate and display the degree of shared coverage of classes/synonyms in comparison to other ontologies?

3. The integration of GO terms into the ontologyt is a nice idea, but it is not clear how deeply they are integrated with the anatomical classes. Please provide more details on this aspect, e.g., by showcasing an example of GO-term enrichment in anatomical subclasses.

4. The authors state that they reviewed 200 primary research citations from 2005 to 2019 (page 5 line 96-98). Please provide criteria by which these papers were selected and for the vetting of future publications.

B) Community collaboration + interface

5. Via planosphere.stowers.org., the authors make available a range of resources such as PLANA, PAGE, OLS, WebVowl. However, these resources are not intuitively reachable from the main page. Especially for PLANA it is unclear which links lead to the user/query interface of PLANA and which lead to related resources and some of the queries mentioned in the text do not work. The inclusion of a clearly labeled direct link from planosphere.stowers.org to planosphere.stowers.org/ontology would allow easier access to this resource. In addition, 2-3 exemplary query tutorials need to be included in order to help users to understand structuring of queries and data output. An online manual would greatly improve the utility of the resource.

6. A valuable part of any ontology is the connections it generates between a search query and primary research citations. PLANA gives a list of pubmed IDs that are linked to the respective query but does not perform any qualitative assessment of the citations. It would be extremely helpful for users to include an indication of either how often the respective term is used in a citation or for what reason.
This could also be the distinction between “mentioning” of a term and citations that have the respective term as a “main topic”. Similarly, indicating from which citations certain links between classes are derived would highly increase the value of the presented tree of terms. For example, PLANA visualizes that the “gut” develops from the “gamma neoblast” but does not indicate the citation from which this information was inferred. A link between this information and the respective pubmed ID would highly increase the ability of users to access the primary research underlying PLANA.

7. The authors state that community suggestions for adding and curating the database will be evaluated by 2 editors. However, it is not clear which parameters or procedures will be applied. What is the procedure on how to mediate conflicts of opinion/evidences? Have the authors incorporated a procedure for retiring classes (this is especially a concern with the initial Neoblast subpopulation labels that are likely to change. Case in point: “sigma neoblasts” vs. Molinaro et al;. 2016? In light of the above, the authors need to explain how they will apply and expand the testing (using queries) to make sure that added, renamed or removed terms will not introduce inconsistencies to the database.

8. The description of planarian resources in the introduction is focused on the author’s own resources. For the benefit of the readers, they should discuss the utility of PLANA also in the context of the other resources.

Minor points

1. Large sections of the text are written by computer science experts- which is a good thing given the subject of the manuscript. However, editing for clarity by a biologist could make the manuscript and therefore also the tool more accessible to the readership of development.

© 2021. Published by The Company of Biologists under the terms of the Creative Commons Attribution License (https://creativecommons.org/licenses/by/4.0/).

2. Page3, line 46: instead of “visual“ it should be “morphological”
3. Page 5: eye and photoreceptor is not a good example of synonymous terms as eyes in the animal kingdom consist of many photoreceptors.
4. The letters of “nervous system“ and “ventral epidermis” overlap in Figure 3B-3C
5. Page 10: “proto-kidneys” is not a scientific term- please remove.
6. Page 11, Fig. 4D (REFERENCES): The authors need to cite Vu et al. here, as this is the study that defines the term “collecting duct”.
7. Clarify why blat and no other alignment software such as blast was used
8. Similarly, the authors state in the introduction that “no generally agreed upon reference transcriptome is available”, yet later on, they designate their own transcriptome the reference transcriptome. Please clarify.

Reviewer 3

Advance summary and potential significance to field

Nowotarski et al. describes 3 new resources for the planarian community: PLANA, PAGE, and Rosetta Stone.
PLANA is an ontology database of newly curated terms that describe subcellular to systems level anatomical features. PAGE is a search tool that integrates gene expression data with the terms defined by PLANA. As there are different transcriptomic and genomic data from multiple labs, Rosetta Stone is a tool that allows one to cross-reference transcripts from multiple sources. All three are tools with limited but appreciable benefits to the planarian research community.

Comments for the author

These tools are hosted on the site Planosphere, which is run by the authors from the Sanchez lab. The missed opportunity here is that these resources are not integrated with PlanMine: the widely used compilation of genomic and transcriptomic data used by the planarian community at large. PlanMine already has a wealth of gene expression data including changes after perturbations (like RNAi, irradiation injury/regeneration response, etc), changes by axes, changes by biotype, FACs data for neoblasts, and all three major single cell sequencing datasets. Hence, the addition of anatomy-based gene expression searches would be more useful to the community if it were hosted at and integrated into PlanMine. Since this is not the case, the inclusion of Rosetta Stone will be helpful so that anyone who uses PAGE will be able to extract the relevant “dd” transcripts that most planarian researchers use. However, I implore the authors to integrate into PlanMine. If all these different tools were found in one place where and we could all coalesce around using the same transcript IDs that are linked to the genome, this would be an enormous help to the community and the value is each individual tool will be magnified. If for some reason the authors think that there is some advantage to NOT integrating with PlanMine, please address this issue in the manuscript.

As it stands, PAGE allows one to search for transcript expression by 3 categories: PLANA terms, exact transcript ID, and publication by Pubmed ID. I have tried a number of searches on all three pages and the results meet expectations. I particularly like that results can be downloaded as results tables and that the sequence results are easily extracted as FASTA files. In my attempts to cross-check the fidelity of the search results, I attempted to compare results by using the “Gene Search” option on the Planosphere site. This is where I encountered a frustrating number of problems. Gene Search should allow me to search for transcripts by gene name but the search function is highly flawed. Here are some examples where I searched using the “contains any word” option, which should return the broadest number of results:
Search for “catenin” yielded no results
Search for “Tropomyosin” yielded no results even though search for SMED30015345 did find an entry that has the name “Tropomyosin”.
Search for “nanos” yielded no results even though search for SMED30034750 found an entry for nanos-like protein (alias nos).
Search for “collagen” yielded no results and “collagen IV” yielded 3 entries for activin and diversin. This is despite the fact that SMED30001376 for “Collagen IV NCI domain-containing protein” does exist.

© 2021. Published by The Company of Biologists under the terms of the Creative Commons Attribution License (https://creativecommons.org/licenses/by/4.0/).

These problems appear to be related to poor set-up of Planosphere (not PLANA or PAGE). Nonetheless launching these tools on a site so rife with search errors is going to discourage users and will likely reduce their utility. Prior to publication, the authors should fix these problems.
This manuscript makes the case that ontological data is a valuable addition that will be helpful to researchers.

If one were interested to find all the transcripts expressed in the planarian eye, PAGE allows you to perform that search and download results that have been largely manually curated based on published data. The curation is based on laborious efforts on the part of the authors from 200 publications. While this is a service to the community that provides researchers a shortcut, it does also rely on the quality of documentation from the original publications. I have done a few searchers to find tissue-specific genes that were described in previously published works which failed to turn up using PAGE. However, the problems usually stemmed from annotation inconsistencies in the original publications rather than problems with PLANA or PAGE. Given that the utility of this kind of analysis advocated in this manuscript should be better discussed. The manuscript would greatly benefit from a demonstration (case study) of how this kind of analysis compares to other methods, for example relying on single cell sequencing data. In this manuscript, Figures 6 and 7 show screen shots from the website to illustrate the kinds of results one can get from anatomical-based searches. This is highly superficial. The manuscript would greatly benefit from a more thorough demonstration of the application of this resource.

Most of the manuscript is devoted to describing how PLANA was designed, sometimes to excess. More than 3 pages are devoted to describing relation structures, which are perfectly simple and intuitive to understand and are clearly delineated in Table 2. However, there is no analysis of how the current state of published gene expression data corresponds to PLANA terms and where “holes in our knowledge” exist. From the 855 class terms in PLANA, how is the gene expression data skewed? Many of the searches that I performed based on PLANA terms resulted in no hits. Some of this is because of over reliance on RNA-based methodologies in the field (for example, most subcellular compartment terms return no results). Others are because researchers have not devoted enough efforts to describing expression patterns in enough detail to capture the cellular complexity that must exist in the animal. The authors should use this manuscript to demonstrate how PLANA highlights the weaknesses in the existing descriptions of gene expression data. Conversely, the authors could also demonstrate how PLANA could assist researchers to capture a more comprehensive gene expression picture of any given cell type. This would help bolster the case for the value of this resource.

The authors advocate the value of PLANA in promoting a controlled vocabulary to streamline gene expression data with anatomical features. Other than the description of some synonymous terms, the manuscript doesn’t actually present any examples of how they have clarified terminology in the field. There is at least one very glaring example that has been left untouched by this work: the extremely loose and inconsistent use of the terms “stem cells” and “progenitors” by planarian researchers. This manuscript should be the place where clarification is made about how to distinguish these two classes and to dictate how all future research should delineate markers of “stem cells” vs “progenitors”. This would indeed be a valuable contribution of an ontology-gene expression database that could promote better practices by researchers in the field.
According to the reviewer guidelines for Techniques and Resources published in Development, I find that this manuscript does an acceptable job of describing the resource and of providing accessible source data for use and application by others. The resources are on an error-prone site (Planosphere) that requires optimization before the launch. The manuscript does a poor job of describing the value, utility, and application of this resource. In short, without major revisions, I do not recommend this manuscript for publication in Development.

If the authors wish to make changes and resubmit to Development, the following major changes are necessary:
1. Integration into PlanMine would be ideal, but if not, the authors should address what advantage there is to maintaining yet another curated resource site.
2. The Gene Search function on Planosphere is faulty and must be fixed. Users who try to use PLANA and PAGE will find that they cannot confirm their results easily by doing gene name searches on this site, which will discourage usage of all these tools and undermine confidence in the site in general.

© 2021. Published by The Company of Biologists under the terms of the Creative Commons Attribution License (https://creativecommons.org/licenses/by/4.0/).

3. Summarize how the 855 PLANA terms capture the existing gene expression data in the field. How is the data skewed? Where are the data under-represented?
4. Provide examples of how ontological vocabulary has been clarified in this manuscript. In particular address how the terms stem cell vs progenitor will be treated going forward.
5. Describe by way of at least one case study, how these tools that provide anatomy-based gene expression data compare to other methods (eg. single cell sequencing). These analyses could replace the superficial descriptions in Figure 6 and 7.
6. Rewrite the manuscript to emphasize the value and application of this resource. Simplifying description of relationship terms will free-up a lot of space (Table 2 is more than sufficient). Figures 3 and 5 can also be removed to the supplement.

First revision

Author response to reviewers' comments

Response to Reviewers:
We thank you, Dr. Poss, and all three Reviewers for their insightful and constructive feedback on our manuscript DEVELOP/2020/196097, “The Planarian Anatomy Ontology: A resource to connect data within and across experimental platforms” by Nowotarski and colleagues. We appreciated this opportunity to hone our communication about the utility of PLANA as an open-source, extensible framework that can be used by other tools to curate, aggregate and mine data. We revised the manuscript extensively to cater to our target audience: biologists who will interact and use PLANA and PLANA-dependent data organization tools. Through incorporation of reviewer-suggested analysis of benchmarking metrics, we demonstrate that PLANA is comparable to extant anatomy ontologies from other species in scope and specificity. Our analysis also shows that PLANA excels relative to other ontologies in capturing known biological information relating classes to one another, bolstering PLANA’s potential for inference and hypothesis generation. In addition to changes made to the manuscript, we revised and updated ancillary features on planosphere.stowers.org to clarify search functions and returned results, and to improve the user experience when interacting with PLANA-generated tools on this site. We now provide how-to guides and video tutorials to assist users navigating PLANA, as well as the Rosetta Stone and PAGE tools. Our overarching goal has been to maximize the value and utility of PLANA and PLANA-generated tools for consumption and adoption by the wider research community. The reviewers’ comments were instrumental in making needed improvements in our work and we are happy to report that all major and minor concerns have been addressed to our satisfaction. We provide a point-by-point rebuttal to all raised questions and criticisms below.

Reviewer 1 Advance Summary and Potential Significance to Field:
The paper presents an ontology of planarian anatomy and development, as well as a gene expression database structured by this ontology. The source of various components in the ontology pulled from literature and existing ontologies and the process of their assembly are described in detail.
This appears to be a highly valuable resource for at least researchers within this growing field. The description of the process should also be useful to those creating novel ontologies.

Reviewer 1 Comments for the Author:
The presentation is clear and succinct, however given the potential interest to people building related ontologies it might help to expand details in a few places and in general further clarify the motivation for decisions, when they are noteworthy and when they are the most obvious course to at least an experienced ontology user.

Major

Some more detail might be helpful in a couple places:

1) More formally what makes something a ‘top level’ Uberon entry to be imported directly and subject to changes in that ontology and what makes something a lower-level entry that is

© 2021. Published by The Company of Biologists under the terms of the Creative Commons Attribution License (https://creativecommons.org/licenses/by/4.0/).

integrated indirectly as a copy and cross reference? I have the intuition it provides some tradeoff between stability and interoperability, but what does the particular division used achieve exactly and how is it implemented by the dividing line?

Thank you for pointing out that the rationale for direct class imports versus instantiation was not clear in the original submission. We have edited the text to better explain these points as detailed below:

In order to better introduce the concept of interoperability we further clarify/define interoperability in the introduction by the addition of the following sentence (lines 103-105):

> "Interoperability of ontologies is provided through parallel data structures and common annotations allowing systems to mutually exchange and make use of information."

To better explain when we imported Uberon terms we heavily edited these lines of the results section (new line 146-154) which now read:

> "In order to extend the use of PLANA and promote interoperability with other ontologies, high-level classes from the Uberon anatomy ontology hierarchy (Mungall et al., 2012) were imported directly into PLANA (e.g., 'anatomical entity' UBERON:0001062 and 'life cycle stage' UBERON:0000105). These wide-ranging, inclusive classes confer the basic structure and frequently appear as nodes in the PLANA hierarchy (Figure 2B). Imported Uberon classes retained all annotation fields as they appear in their ontology of origin and no additional annotations were added. Importantly, these imported classes are subject to change when Uberon is updated, thus it is important they are sufficiently broad to accurately encompass planarian anatomy. "

To more clearly explain when/why a term is instantiated we largely revised the following paragraph (lines 155-170):

> "To facilitate comparative anatomy queries across species, additional classes from extant anatomy ontologies were instantiated into PLANA whenever possible, including the Common Anatomy Reference Ontology (CARO) (Haendel et al., 2008), the Biological Spatial Ontology (BSPO) (Dahdul et al., 2014), the Cell Ontology (CL) (Diehl et al., 2016), the Gene Ontology (GO) (Ashburner et al., 2000; The Gene Ontology Consortium, 2019) and Uberon (Mungall et al., 2012). Instantiation imports a class and allows modifications and additions to class annotations to accurately reflect planarian-specific anatomical information. Importantly, annotations associated with instantiated classes do not change when their ontology of origin is updated. For example, the class 'eye' UBERON:0000970 was instantiated into PLANA so that it could be annotated with information about its cellular origin, development, and anatomical location in planarians. Instantiated terms were assigned a new PLANA id (e.g., 'eye' PLANA:0000036), but the original external ontology identification number was retained in the database cross-reference (dbxref) annotation field so analogous terms remain associated. Class instantiation promotes interoperability of ontologies by ensuring that analogous terms are findable (indexable), associated, and yet remain tailored to planarian anatomy."

2) Is there a specific strategy or rule use in applying auto composite to create entries or is this ad-hoc?

Composite terms were created ad-hoc. More specifically, the strategy or rule is if we can create a patterned phrase that can be used to predictably and systematically generate a collection of classes (more than 1) we use composite terms. This is to enforce findability of classes and reuse of classes. We have made this clearer in the text by adding to and editing the following (lines 208-213) to read:

> "During the literature survey to generate class names (Table S1), multi-word classes were included (e.g., 'photoreceptor neuron'). However, the need to create multiple terms that follow a common pattern became apparent (e.g., 'anterior photoreceptor neuron' and 'posterior photoreceptor neuron'). It became clear that many classes could be created as needed using an additive, formulaic approach already employed by other ontologies. "

© 2021. Published by The Company of Biologists under the terms of the Creative Commons Attribution License (https://creativecommons.org/licenses/by/4.0/).

3) From examples it seems like explicit use of transitive relationships is used minimally to create appropriate connected components with all nodes appropriately reachable. This seems like a logical strategy, but it's not explicitly stated, is it the case? If not, what are exceptions?

This is true, the transitivity of relationships allows us to use minimal connections to achieve maximum connection. We have now made this explicit in lines 236-237:

"To maximize inference from minimal information we assigned transitive relationships spanning one level of anatomical organization."

Minor

Couple other points of minor clarification:

4) When table 2 is referenced it might increase clarity to note its contents if it does not appear on the same page, it was unclear it contained the transitivity information from the text.

We agree that this would be best, but because we cannot know the final formatting for publication we cannot predict where the reference for Table 2 will be. However, we have changed the title of Table 2 to read:

"Table 2: Object Property Relationship Terms. Ontological definitions of each object property, realm, transitivity, description of rules for application in PLANA, and an example of use in PLANA."

5) I presume the properties of all the relations in this table are derived directly from the Relational Ontology, if not maybe this should be clarified.

This is correct and thank you for pointing out that this was not explicit in the text. It has been clarified in the text (lines 225-228):

"This release of PLANA uses 14 object properties, all from the Relationship Ontology (RO)(Smith et al., 2005), which enable the construction of categorical, spatial, developmental, and temporal relationships between classes (Table 2, Materials and Methods)."

6)It is worth noting if depicted by is a single-entry field or if multiple illustrations of an entry are possible in theory.

We clarified this in the text with the additions of the sentence in in lines 143 & 144:

"For each annotation field, multiple entries are permitted."

Reviewer 2 Advance Summary and Potential Significance to Field:
The manuscript by Nowotarski et al. endeavors to establish an anatomical ontology for S. mediterranea, both as a resource to the planarian research community and as an interface with other research communities. The authors align their efforts along the published ontology recommendations by Druzinsky et al. (which is cited in the text) and they make the ontology accessible via their own web resource.
Overall, this manuscript is clearly based on a multi-year effort and as stated by the authors, an anatomical ontology could indeed amount to a valuable and timely resource for the field. Moreover, the authors make provisions for community involvement, including for example a useful feedback submission procedure using the Github platform. As such, the manuscript is a suitable contribution to the "resource section" of Development. That said, concerns remain regarding the benchmarking and accessibility of the resource (see major points below). Although the authors clearly state that "…PLANA does not claim to be comprehensive or exact", they also state that they see PLANA "As a standard for anatomical information". Our major points pertain to the establishment of said standard.

Reviewer 2 Comments for the Author:

Major Points

A) Benchmarking

© 2021. Published by The Company of Biologists under the terms of the Creative Commons Attribution License (https://creativecommons.org/licenses/by/4.0/).

1. The authors need to compare their de novo established ontology against existing ontologies of other model systems. Metrics for the comparisons should include the number of classes and the granularity or number of child terms originating from the parent terms. This is important in order to gauge the breadth and depth of the ontology.

We appreciate the feedback to contextualize PLANA within the field of extant ontologies. While ontologies strive for appropriate coverage reflecting current use, knowledge, and application within the field, we understand that reporting benchmarking metrics would provide an objective measure by which to evaluate PLANA with respect to its peers. Favorable performance of PLANA relative to other anatomy ontologies will assure prospective users that PLANA is a high-quality framework worthy of use and continued investment.

There are a number of factors that play into class number and coverage for an ontology: organismal complexity, completeness of the existing knowledge base, adoption by stakeholders, and research efforts targeted at specific anatomical regions. A good example of this is the *Drosophila* Anatomy Ontology (FBbt). *Drosophila* has a long history as an established research model, a large population of active researchers, and perhaps a more complex anatomy (in current understanding) compared to *Smed*. All of these factors are likely reflected in FBbt having 20x the number of classes as PLANA (17484 to 856). In addition, recent large-team initiatives to map the fly brain at high resolution have led to heavy investment and representation in developing nervous system subclasses in FBbt. Currently, 61% of all classes in FBbt (10839 of 17484) represent nervous system subclasses. In contrast, the nervous system is represented by 7.6% of all classes (65/856) in PLANA. This stark difference is not indicative of a deficit in PLANA relative to FBbt, but rather illustrates how instrumental the research community is in shaping how an ontology evolves and where further granularity is needed to achieve research objectives.

These caveats on composition aside, it is helpful for readers to know both the class number and the scale range of terms associated with currently used anatomy ontologies for established research organisms like Xenopus, Zebrafish and C. elegans. To that end, we have determined class number and the range of classes captured for existing anatomy ontologies and PLANA and present these data in a new figure (Supplemental Figure 1). PLANA and other ontologies use organism-level classes at the top of their hierarchies. Most anatomy ontologies have only cell type level classes represented at their most granular level. Only Xenopus' anatomy ontology includes a full set of subcellular organelles and structures like PLANA. Again, this granularity reflects current and projected uses, where we will be using PLANA to tag high resolution EM images. Strikingly, PLANA outperforms existing anatomy ontologies with respect to relational statements between classes (Supplemental Figure 1C). This metric suggests that PLANA more thoroughly captures existing anatomical information than ontologies for established research organisms and suggests that PLANA's powers of inference will maximize users' abilities to glean novel information and develop new hypotheses using PLANA and PLANA-derived tools.

In summary, the function and applied use by stakeholders largely sets an ontology's form. We have added the following passage with respect to these new Benchmarking metrics (lines 265-279):

"To ensure comparable coverage of PLANA with respect to other anatomy ontologies we checked multiple metrics for benchmarking. Like other anatomy ontologies, PLANA's broadest class starts at the organism level ('whole organism' PLANA:0000136), and its most granular classes are subcellular components (e.g., organelles (Fig. S1A). Comparison of class number between anatomy ontologies reflects differences in (1) gross anatomy between species, (2) depth of current anatomical research and (3) and field-specific use (i.e. neuronal mapping in *Drosophila)*. As expected, given these caveats, PLANA (862) contains more classes than the Ctenophore (646), Tick (629), Echinoderm (497) and Dictyostelium (134) anatomy ontologies, and less than Mouse (adult:3257, development:8643), Xenopus (1763), Zebrafish (3219), and Drosophila (17484) (Fig. S1A). Notably, 61% of Drosophila anatomy ontology classes are nervous system components, a feature that reflects its usage to curate high resolution maps of the fly nervous system (Fig. S1A). Despite PLANA's smaller class count, it's relative complexity and deployment of relationships (axioms/class) is, in fact, greater than the heavily used Uberon, CL and Drosophila ontologies (Fig. S1C)."

© 2021. Published by The Company of Biologists under the terms of the Creative Commons Attribution License (https://creativecommons.org/licenses/by/4.0/).

2. Also, it would be useful to provide the readers with an impression of the interoperability with other ontologies, i.e. the PLANA term “epidermis” is missing the synonym 'skin'.

We agree that this is a good idea and have added a diagram detailing “homology”, or equivalence between classes, using ‘basal lamina of epithelium’ class as an example in Supplemental Figure 3A. We added the following text (lines 294-297) to the manuscript:

“Interoperability is also provided by including “homologous” classes from other anatomy ontologies in the dbxref annotation, for example ‘basal lamina of epithelium has 15 “homologous” classes (Table1; Fig. S3A).”

With respect to ‘skin’, it was not a synonym for ‘epidermis’ in the publications queried in our literature search (Table S1). As a rule, we don’t create synonyms that aren’t in use by the field. We have clarified this rule in the text (lines 179 & 180):

“Classes may be annotated with multiple synonyms (Figure 2A) and synonyms for this release were only taken from the literature search (Table S1).”

3) Is there a way to evaluate and display the degree of shared coverage of classes/synonyms in comparison to other ontologies?

In order to address this, we compared class to synonym ratio per ontology in Supplemental Figure 2A, and assessed direct classes/synonym overlap using a Venn diagram (Supplemental Figure 2B). This information is included in the text (lines 279-282):
“Additionally, the relative ratio of synonyms to classes is equivalent to other ontologies (0.72 average synonyms per class; Fig. S2A). A third of PLANA (34.2%) class labels and synonyms corresponds to those of highly-used anatomy ontologies (Fig. S2B).”

4). The integration of GO terms into the ontology is a nice idea, but it is not clear how deeply they are integrated with the anatomical classes. Please provide more details on this aspect, e.g., by showcasing an example of GO-term enrichment in anatomical subclasses.

In PLANA, GO terms are associated with classes in two ways. First, GO terms appear in the dbxref field for GO terms instantiated into PLANA (e.g., microvillus, cell, cell projection). Instantiation allowed us to modify the terms as necessary but kept record of the ontology of origin in the dbxref field, facilitating interoperability of ontologies. Second, a select set of GO terms were imported in order to create more specific anatomical classes such as ‘metaphase neoblast’ PLANA:0003204 (‘mitotic metaphase’ GO:0000089 + ‘neoblast’ PLANA: 0000429) through composite patterning. Therefore, our use of GO terms in PLANA does not signify GO enrichment per se. We have included all GO Terms used in PLANA in Supplemental Table 5. We have also made this use clear in the interoperability section (lines 307-309):

“At present 160 GO terms are used in PLANA and they largely occupy organelle level classes and cell cycle phases (Table S5).”

Outside of PLANA, we have addressed GO enrichment associated with transcripts both included and not included in the PAGE resources per Reviewer 3’s requests, see below for more information.

5. The authors state that they reviewed 200 primary research citations from 2005 to 2019 (page 5 line 96-98). Please provide criteria by which these papers were selected and for the vetting of future publications.

Thank you for pointing out this oversight on our part. This information now appears in the Supplemental Table 1 Legend. We also include the Literature Search parameters in the main text of the Materials and Method Section lines (523-529):

“The primary literature search was carried out in PubMed (search terms: planaria, Smed, Schmidtea mediterranea) and papers were not included if they were from species other than Smed. Papers from 2005 (advent of large screens) - 2019 and a few landmark works prior to that period

© 2021. Published by The Company of Biologists under the terms of the Creative Commons Attribution License (https://creativecommons.org/licenses/by/4.0/).

were included; reviews and protocol papers were excluded. A few papers were Smed-specific, but contained either no anatomical terms or very few anatomical terms that were broad and redundant (e.g. head) and those papers were not included."

We also added these requirements to the MIRO reporting (Supplemental Table 13) in D1: Knowledge Gathering which now reads:

"**Gathered:** Three domain experts scoured primary literature for anatomical feature terms to become classes. The primary literature search was carried out in PubMed (search terms: planaria, Smed, *Schmidtea mediterranea)* and papers were not included in the survey if they were from species other than Smed. Papers from 2005(advent of large screens) - 2019 were included. A few papers were Smed specific, but contained either no anatomical terms or few anatomical terms that were broad and redundant (i.e., head). Those papers did not get included. For future updates past 2019 the same search parameters will be put into PubMed and same selection criteria will be used (must be Smed only). In addition, we used extant terms from other ontologies (BSPO, GO, PATO, RO, UBERON) for both organization and terms. If a term from another ontology had a definition that was appropriate, it was kept. If the term required a change to make it planarian specific that extant term was included as a dbxref."

B) Community collaboration + interface

5.Via planosphere.stowers.org., the authors make available a range of resources such as PLANA, PAGE, OLS, WebVowl. However, these resources are not intuitively reachable from the main page. Especially for PLANA it is unclear which links lead to the user/query interface of PLANA, and which lead to related resources and some of the queries mentioned in the text do not work. The inclusion of a clearly labeled direct link from [planosphere.stowers.org]planosphere.stowers.org to [planosphere.stowers.org/ontology]planosphere.stowers.org/ontology would allow easier access to this resource

Thank you for your feedback on website design. we have added the direct link Reviewer 2 suggested and have also added links specific to PLANA browsing in the "Tools" header menu.

6) In addition, 2-3 exemplary query tutorials need to be included in order to help users to understand structuring of queries and data output.

We agree that tutorial videos are needed. We now have 14 tutorials available on Planosphere. All tutorials are available at https://planosphere.stowers.org/allresources/tutorials (accessible through the HELP menu link), and through links on resource pages. Tutorials cover the following topics:

- What is PLANA?
- Interacting with PLANA on OLS
- Interacting with PLANA on Planosphere
- Accessing PLANA
- About Relationships in PLANA
- Request a change to a PLANA Class
- How to submit a New PLANA Class
- How to submit a bulk New Class Request
- How to use Rosetta Stone Transcript Mapper on Planosphere
- What is PAGE?
- How to run a PAGE Transcript query
- How to run a PAGE Anatomical query
- How to run a PAGE Publication query
- Navigation of Transcript Pages

7) An online manual would greatly improve the utility of the resource.

Because Planosphere serves as a resource and data aggregator for Sánchez Alvarado lab publications, it's not meant to act as a completely integrated unit. As such, there is no prescribed

© 2021. Published by The Company of Biologists under the terms of the Creative Commons Attribution License (https://creativecommons.org/licenses/by/4.0/).

workflow that takes a user from tool to tool. To clarify what each resource does and direct users to what they need, we created a "User Guide" page that you can link to from on the homepage in two locations ("Help" and "Not sure what you need?").

The "User Guide" contains a table detailing What, Where, and Why information associated with each resource on Planosphere, along with direct links out. It also includes a link to the above tutorials. The "How to Use" sections remain in the respective resource pages for easy reference when they are needed, again with a link out to the tutorial videos. To better explain how to use each resource we have expanded our written instructions and tips under expandable buttons on each site directly associated with this publication. For an example, please visit PAGE search by Term here: https://planosphere.stowers.org/search/page/term and click on "About this Search" & "Search Tips".

6.A valuable part of any ontology is the connections it generates between a search query and primary research citations. PLANA gives a list of PubMed IDs that are linked to the respective query but does not perform any qualitative assessment of the citations. It would be extremely helpful for users to include an indication of either how often the respective term is used in a citation or for what reason. This could also be the distinction between "mentioning" of a term and citations that have the respective term as a "main topic".

While we agree with Reviewer 2 that this would-be useful information it is beyond the scope of our work. Anatomy ontologies are built to hold qualitative data rather than quantitative data. Specifically, anatomy ontologies complying with OBO Foundry and that are interoperable with Uberon do not have instance information attached to them, which would be required to hold the numerical data the Reviewer is suggesting. However, the PAGE database already has publication information embedded into it and users can look there for it. The advantage of a publication showing up in PAGE associated with a PLANA term(s) is that it is inherently more "main topic" by virtue of carrying expression information rather than just an in-passing mention in text. In addition to anatomy ontologies not holding this type of data, and PAGE carrying some of it, we were also concerned that an unbiased text search that quantifies class use would not be sufficient to identify the 'main topic' of a paper. Classes that codify important anatomical landmarks, e.g., 'cephalic ganglia' or 'pharynx', appear frequently in papers that are not focused on brain/pharynx development, or brain/pharynx regeneration per se. This type of analysis may be possible to automate in the future, as text search and ontology interface tools become better at mining information for context, not simply information content. This is a space we are actively watching for future applications.

Similarly, indicating from which citations certain links between classes are derived would highly increase the value of the presented tree of terms. For example, PLANA visualizes that the "gut" develops from the "gamma neoblast" but does not indicate the citation from which this information was inferred. A link between this information and the respective pubmed ID would highly increase the ability of users to access the primary research underlying PLANA.

This information is already provided in PLANA, built into the def_dbxref annotation property. The def_dbxref annotation is used in PLANA to provide provenance for definition and relationships used for terms. For example, 'gamma neoblast' PLANA:0000039, holds that particular relationship information in the def_dexref annotation field with PMID: 25017721 (van Wolfswinkel et al., 2014). We have made this connection clearer in the text (lines 137-139):

"Each class was annotated with required information as follows: a singular name (label), e.g., 'eye', a unique identification number (ID), a definition, and the relevant reference(s) for the definition and the relationships for each class (def_debxref, Figure 2A)."

7. The authors state that community suggestions for adding and curating the database will be evaluated by 2 editors. However, it is not clear which parameters or procedures will be applied. What is the procedure on how to mediate conflicts of opinion/evidences? Have the authors incorporated a procedure for retiring classes (this is especially a concern with the initial Neoblast subpopulation labels that are likely to change. Case in point: "sigma neoblasts" vs. Molinaro et al;. 2016? In light of the above, the authors need to explain how they will apply

© 2021. Published by The Company of Biologists under the terms of the Creative Commons Attribution License (https://creativecommons.org/licenses/by/4.0/).

and expand the testing (using queries) to make sure that added, renamed or removed terms will not introduce inconsistencies to the database.

As is true for any open source and editable tool, the shape and direction of future growth will be driven by the stakeholders who use PLANA. Changes will be driven by deposition of terms, and making change requests, which are all done through the Issue Tracker on GitHub (https://github.com/obophenotype/planaria-ontology/issues). To participate in discussions around additions, changes, and updates to existing PLANA content, stakeholders simply "watch" the GitHub Issues page and receive email notifications that alert them to all proposed changes as they come up. Any interested member of the planarian research community can "watch' the Issue Tracker and participate in discussions about PLANA updates.

We provide additional information about the vetting process for proposed changes to PLANA (lines 446-470):

"Members of the research community are encouraged to assist with PLANA curation through submission of a new class(es) and/or proposing edits to an existing class(es) using the GitHub issue tracker (https://github.com/obophenotype/planaria-ontology/issues). New class submissions require a class name, definition, PMID or DOI numbers for publication(s) referencing the definition, and a contact name and email address for the contributor. Two curators will review new classes and other proposed edits and will correspond with the contributor to resolve outstanding questions prior to updating PLANA. Bulk requests for new classes should be submitted using the spreadsheet template posted on the PLANA GitHub issue tracker. Requests without evidence (publication id) upon submission will not be accepted.

When issues consist of changes that are unanimously agreed to by the reviewers and have a citation for supporting evidence, the changes and/or insertions will be made after a minimum of 1 week waiting period. This waiting period is used to monitor discussions for and against the proposed changes in the GitHub issue forum. If no discussion is created on a pending issue, the changes will be made as per the request. When proposed changes, with published evidence, generate contradictory discussions within the 1 week waiting period, the arguments will be evaluated by a third researcher in the field (requested via email) and a decision by the new reviewer will be made, with discussion and reasoning posted on the Issue Tracker page. If the reviewers cannot agree, and a long term solution cannot be agreed upon, the issue will be addressed in a session at the next International Planarian Meeting. Following and participating in the discussions on proposed changes to PLANA is open to all, and only requires users to sign up for a free GitHub account and "watch" the PLANA repository. Watchers will receive email notifications when issues and discussions arise and when PLANA is updated."

8. The description of planarian resources in the introduction is focused on the author's own resources. For the benefit of the readers, they should discuss the utility of PLANA also in the context of the other resources.

Thank you for making this point. We have explicitly pointed out a recent use of PLANA outside of the Sánchez Alvarado lab and publication in lines (507 & 508):

"In addition to PAGE, PLANA has been used to annotate personally-curated, image-based, expression patterns in PlanGexQ(Roy et al., 2020)."

In addition, we now point out examples of how extant anatomy ontologies are used. Established use cases for other anatomy ontologies suggest how PLANA may be used in the future (lines 96-97):

"These ontologies are integral for field resources such as the Virtual Fly Brain (Milyaev et al., 2012; Osumi-Sutherland et al., 2014). "

Minor points

1. Large sections of the text are written by computer science experts- which is a good thing given the subject of the manuscript? However, editing for clarity by a biologist could make the manuscript and therefore also the tool more accessible to the readership of development.

© 2021. Published by The Company of Biologists under the terms of the Creative Commons Attribution License (https://creativecommons.org/licenses/by/4.0/).

Thank you for this important feedback. We rewrote and reorganized the manuscript to address your concerns regarding the technicality of the text. Importantly, we removed the lengthy portion of the Results section detailing precise rules for use of relationships. This information is now condensed and largely represented in Table 2.

2. Page3, line 46: instead of "visual "it should be "morphological "

This has been replaced as suggested.

3. Page 5: eye and photoreceptor is not a good example of synonymous terms, as eyes in the animal kingdom consist of many photoreceptors.

Once again, this is great feedback. We personally agree with you that eye and photoreceptor are not strict anatomical synonyms, but we found in our literature review that planarian researchers often (and erroneously) use these terms interchangeably. In the text we make this rule clear now (lines 179 - 180):

"Classes may be annotated with multiple synonyms (Figure 2A) and synonyms for this release were only taken from the literature search (Table S1)."

4. The letters of "nervous system" and "ventral epidermis" overlap in Figure 3B-3C

Thank you for pointing this out. We have rectified this error.

5. Page 10: "proto-kidneys" is not a scientific term- please remove.

This phrasing had been changed from "in the proto-kidneys" to: " In the kidney units," and then was ultimately cut in favor removing overly technical portions of the text and per your and Reviewer 3's suggestions.

6. Page 11, Fig. 4D (REFERENCES): The authors need to cite Vu et al. here, as this is the study that defines the term "collecting duct".

We are sorry to have left this citation out, thank you for pointing out this oversight. The citation was added as suggested, but ultimately this section of text was cut in favor removing overly technical portions of the text and per Reviewer 3's suggestions.

7. Clarify why blat and no other alignment software such as blast was used

Though BLAST offers greater flexibility and sensitivity for evolutionarily divergent sequences, we are aligning high-identity nucleotide sequences from the same species. We opted to use BLAT, a proven and well-established algorithm used by default by both Ensembl and UCSC for nucleotide alignments. Other long read aligners like BLAST or FASTA would be expected to produce nearly identical results.

8. Similarly, the authors state in the introduction that "no generally agreed upon reference transcriptome is available", yet later on, they designate their own transcriptome the reference transcriptome. Please clarify.

We do not mean to place value judgements on transcriptome choice, nor do we mean to imply that our transcriptome of choice, smed20140614, should be adopted as a reference standard for the field at large. We removed the first instance of this idea in the introduction, which was made in reference to awaiting gene models for the *Smed* genome (Grohme et al., 2018). Right now, folks make do within their respective labs with their favorite or best transcriptomes for their respective tools and we've done the same. Removing that first phrase from the manuscript should remove any perceived stance on transcriptome hierarchy, which was not our intent. To that end, we have altered the second phrasing to remove "reference" and added a sentence to make it clear that PLANA is reference-independent and can work with any transcriptome.

The passage now reads (396-403):

© 2021. Published by The Company of Biologists under the terms of the Creative Commons Attribution License (https://creativecommons.org/licenses/by/4.0/).

"Because accessions and identifiers for annotations came from multiple transcriptomes and other sources like ESTs, we built a translation tool, Rosetta Stone Transcript Mapper, to map all sequences back to the smed_20140614 ~~reference~~ transcriptome (https://planosphere.stowers.org/search/rosettastone/blaze; Suppl Figure 1)(Tu et al., 2015). Although any transcriptome could have been selected as a reference, the smed_20140614 transcriptome was used because it is currently the transcriptome of choice for next-generation sequencing mapping in our laboratory. PLANA itself is a reference-independent tool, and can be used in conjunction with any transcriptome."

Reviewer 3 Advance Summary and Potential Significance to Field:
Nowotarski et al. describes 3 new resources for the planarian community: PLANA, PAGE, and Rosetta Stone.PLANA is an ontology database of newly curated terms that describe subcellular to systems level anatomical features. PAGE is a search tool that integrates gene expression data with the terms defined by PLANA. As there are different transcriptomic and genomic data from multiple labs, Rosetta Stone is a tool that allows one to cross-reference transcripts from multiple sources. All three are tools with limited but appreciable benefits to the planarian research community.

Reviewer 3 Comments for the Author:
These tools are hosted on the site Planosphere, which is run by the authors from the Sanchez lab. The missed opportunity here is that these resources are not integrated with PlanMine: the widely used compilation of genomic and transcriptomic data used by the planarian community at large. PlanMine already has a wealth of gene expression data including changes after perturbations (like RNAi, irradiation,injury/regeneration response, etc), changes by axes, changes by biotype, FACs data for neoblasts, and all three major single cell sequencing datasets. Hence, the addition of anatomy-based gene expression searches would be more useful to the community if it were hosted at and integrated into PlanMine. Since this is not the case, the inclusion of Rosetta Stone will be helpful so that anyone who uses PAGE will be able to extract the relevant "dd" transcripts that most planarian researchers use. However, I implore the authors to integrate into PlanMine. If all these different tools were found in one place where and we could all coalesce around using the same transcript IDs that are linked to the genome, this would be an enormous help to the community and the value is each individual tool will be magnified. If for some reason the authors think that there is some advantage to NOT integrating with PlanMine, please address this issue in the manuscript.

As it stands, PAGE allows one to search for transcript expression by 3 categories: PLANA terms, exact transcript ID, and publication by Pubmed ID. I have tried a number of searches on all three pages and the results meet expectations. I particularly like that results can be downloaded as results tables and that the sequence results are easily extracted as FASTA files. In my attempts to cross-check the fidelity of the search results, I attempted to compare results by using the "Gene Search" option on the Planosphere site. This is where I encountered a frustrating number of problems. Gene Search should allow me to search for transcripts by gene name but the search function is highly flawed. Here are some examples where I searched using the contains any word" option, which should return the broadest number of results:

Search for "catenin" yielded no results

Search for "Tropomyosin" yielded no results even though search for SMED30015345 did find an entry that has the name "Tropomyosin".

Search for "nanos" yielded no results even though search for SMED30034750 found an entry for nanos-like protein (alias nos).

Search for "collagen" yielded no results and "collagen IV" yielded 3 entries for activin and diversin. This is despite the fact that SMED30001376 for "Collagen IV NCI domain-containing protein" does exist.

These problems appear to be related to poor set-up of Planosphere (not PLANA or PAGE). Nonetheless,launching these tools on a site so rife with search errors is going to discourage users and will likely reduce their utility. Prior to publication, the authors should fix these problems.

© 2021. Published by The Company of Biologists under the terms of the Creative Commons Attribution License (https://creativecommons.org/licenses/by/4.0/).

This manuscript makes the case that ontological data is a valuable addition that will be helpful to researchers. If one were interested to find all the transcripts expressed in the planarian eye, PAGE allows you to perform that search and download results that have been largely manually curated based on published data. The curation is based on laborious efforts on the part of the authors from 200 publications. While this is a service to the community that provides researchers a shortcut, it does also rely on the quality of documentation from the original publications. I have done a few searchers to find tissue-specific genes that were described in previously published works which failed to turn up using PAGE. However, the problems usually stemmed from annotation inconsistencies in the original publications rather than problems with PLANA or PAGE. Given that, the utility of this kind of analysis advocated in this manuscript should be better discussed. The manuscript would greatly benefit from a demonstration (case study) of how this kind of analysis compares to other methods, for example relying on single cell sequencing data. In this manuscript, Figures 6 and 7 show screen shots from the website to illustrate the kinds of results one can get from anatomical-based searches. This is highly superficial. The manuscript would greatly benefit from a more thorough demonstration of the application of this resource.

Most of the manuscript is devoted to describing how PLANA was designed, sometimes to excess. More than 3 pages are devoted to describing relation structures, which are perfectly simple and intuitive to understand and are clearly delineated in Table 2. However, there is no analysis of how the current state of published gene expression data corresponds to PLANA terms and where "holes in our knowledge" exist. From the 855 class terms in PLANA, how is the gene expression data skewed? Many of the searches that I performed based on PLANA terms resulted in no hits. Some of this is because of over reliance on RNA-based methodologies in the field (for example, most subcellular compartment terms return no results). Others are because researchers have not devoted enough efforts to describing expression patterns in enough detail to capture the cellular complexity that must exist in the animal. The authors should use this manuscript to demonstrate how PLANA highlights the weaknesses in the existing descriptions of gene expression data. Conversely, the authors could also demonstrate how PLANA could assist researchers to capture a more comprehensive gene expression picture of any given cell type. This would help bolster the case for the value of this resource.

The authors advocate the value of PLANA in promoting a controlled vocabulary to streamline gene expression data with anatomical features. Other than the description of some synonymous terms, the manuscript doesn't actually present any examples of how they have clarified terminology in the field. There is at least one very glaring example that has been left untouched by this work: the extremely loose and inconsistent use of the terms "stem cells" and "progenitors" by planarian researchers. This manuscript should be the place where clarification is made about how to distinguish these two classes and to dictate how all future research should delineate markers of "stem cells" vs "progenitors". This would indeed be a valuable contribution of an ontology-gene expression database that could promote better practices by researchers in the field.

According to the reviewer guidelines for Techniques and Resources published in Development, I find that this manuscript does an acceptable job of describing the resource and of providing accessible source data for use and application by others. The resources are on an error-prone site (Planosphere) that requires optimization before the launch. The manuscript does a poor job of describing the value, utility, and application of this resource. In short, without major revisions, I do not recommend this manuscript for publication in Development.

Major Changes

If the authors wish to make changes and resubmit to Development, the following major changes are necessary:

1. Integration into PlanMine would be ideal, but if not, the authors should address what advantage there is to maintaining yet another curated resource site.

We wholeheartedly agree that PlanMine is an invaluable resource for the planarian research community. We fully support incorporation of PLANA, Rosetta Stone Transcript Mapper, and PAGE into PlanMine *post-publication*. Unlike more established research communities, like *Drosophila,* we lack an independent consortium that curates and hosts community resources. Mirroring PLANA on two independent sites, Planosphere and PlanMine, will promote FAIR (findable, accessible,

© 2021. Published by The Company of Biologists under the terms of the Creative Commons Attribution License (https://creativecommons.org/licenses/by/4.0/).

interoperable and reproducible) practices (Wilkinson et al., 2016). Because the authors do not have administrative privileges on PlanMine, mirroring PLANA or making API calls to our data will ensure that the authors retain maintenance privileges while also promoting use and visibility of PLANA on PlanMine. We will be very glad to work with PlanMine administrators to integrate PLANA and PLANA-based tools onto their site.

2. The Gene Search function on Planosphere is faulty and must be fixed. Users who try to use PLANA and PAGE will find that they cannot confirm their results easily by doing gene name searches on this site, which will discourage usage of all these tools and undermine confidence in the site in general.

We thank Reviewer 3 for their diligent testing and feedback about the Gene Search function on Planosphere. We made substantive changes to the Gene Search tool to clarify its output and to make the tool more user-friendly. The "Search by Name" function was not working well, in large part because gene/transcript names are frequently not associated with transcript identifiers and NCBI accession numbers. Manual curation of all names and aliases for smed20140614 transcripts would be too time consuming for us to undertake as part of this work. Instead, we modified Gene Search by requiring queries to use SMED300XXXXX transcript identifiers only. If users do not have SMED300XXXXX ID(s), they may use the Rosetta Stone Transcript Mapper or the BLAST tool to retrieve them. We have also updated and included more documentation on the Gene Search Page that explain how the search works, how it was built, and what a user can expect from it as a tool, both in the header and the linked "About this Gene Search" page (https://planosphere.stowers.org/search/gene/about).

3. Summarize how the 855 PLANA terms capture the existing gene expression data in the field. How is the data skewed? Where are the data under-represented?

We appreciate Reviewer 3's interest in how PAGE data are distributed over the PLANA classes, and present that analysis in Supplemental Table 5 and its associated text. PAGE encompasses data for 19.6% of current PLANA classes. Two factors likely drive this finding: 1) Some of PLANA's parent classes are organizational (e.g.'anatomical entity', 'acellular anatomical structure'), and 20% of our classes are immaterial (axes/planes/cavities) or subcellular components (e.g., organelles). These classes were not expected to capture expression data. 2) The data captured in PAGE reflects author-reported statements about gene expression, and curators did not infer or enter additional information about expression patterns or digital gene enrichment beyond what was stated in the publication. As a result, PLANA classes represented in PAGE range from cell type to anatomical systems and body regions. We've included that information in the following text (lines 408-410):
"PAGE covers 19.6% (168) of PLANA anatomical terms that largely encompass body region, cell and tissue level terms (Table S6)."

Of course, the opportunity for increased granularity within PAGE is possible providing that authors curate their data exhaustively when contributing to the database. For example, rather than simply saying that transcript X is expressed in the 'testis,' the authors could indicate which cell type(s) within the testis express transcript X.

We determined the percentage of PLANA classes, transcripts, and gene models represented in the PAGE database, as well as the breakdown in types of expression evidence across PAGE. These results are included in the text (lines 423-436):

"Aside from providing individual records of expression, PAGE provides a platform to survey the expression landscape. As expected, the majority of the expression information in PAGE was obtained using high throughput methods: single cell RNA Seq (48.3%, 20,089 entries) and RNA-Seq (34.1%, 14,185 entries). Colorimetric and fluorescent in situs, and cDNA microarrays make up the remainder of PAGE database entries with 9.1%(3,774 entries),1.4%(570 entries), and 7.1% (2,929 entries) respectively. PAGE has at least one instance of evidence recorded for 49.6% of the smed_20140614 transcriptome and 67.1% of the smesg gene models."

We ran GO enrichment on transcripts that were 1) included in PAGE or 2) excluded from PAGE. The vast majority of GO Terms are associated with transcripts included in PAGE, and the terms with the highest p value clearly represent active areas of research for the *Smed* community.

© 2021. Published by The Company of Biologists under the terms of the Creative Commons Attribution License (https://creativecommons.org/licenses/by/4.0/).

GO Enrichment for PAGE-excluded transcripts returned a large amount of viral terms, and topics not frequently studied in the field. We've added this information into the manuscript in Table 3 and the following passage (lines 429-435):

"GO enrichment of transcripts recorded in PAGE returned 8032 terms and revealed that the top three GO terms by corrected p-value were: cell differentiation, cellular developmental process, and animal organ development (Table 3; Table S8,9). GO Enrichment of transcripts not included in PAGE cover only 364 GO Terms with the top three by corrected p-value being: DNA integration, transposition, and catalytic activity, acting on DNA (Table 3; Table S10,11)."

4. Provide examples of how ontological vocabulary has been clarified in this manuscript. In particular, address how the terms stem cell vs progenitor will be treated going forward.

The purview of an ontology is to document, as comprehensively possible, an existing domain of knowledge. PLANA seeks to capture all anatomical terms used by planarian researchers. To this end, we conducted a literature search (Table S1) to identify needed classes, synonyms (to allow for variability in language used by different researchers in the field), term definitions, and supporting information. The ontology creators, and the consortium of curators that will be assembled in the future through community involvement, do not seek to unilaterally decide and impose language choices for the field. Such decisions are made according to precedent from published work, as well as community discussions that occur through the Github Issue Tracker. PLANA is a living document whose classes and relationships are meant to evolve with time to reflect new and changing knowledge. We address the evolving nature of PLANA in the following lines (439-441):

"PLANA is a living resource. Changes to PLANA will be made by the manuscript authors and stakeholders in the community to reflect advances reported in future publications."

We establish and explain the process for addition of new classes and relationships, as well as how changes to existing classes will be made in the future, including definition changes and deprecation of terms and/or synonyms (lines 456-470):

"When issues consist of changes that are unanimously agreed to by the reviewers and have a citation for supporting evidence, the changes and/or insertions will be made after a minimum of 1 week waiting period. This waiting period is used to monitor discussions for and against the proposed changes in the GitHub issue forum. If no discussion is created on a pending issue, the changes will be made as per the request. When proposed changes, with published evidence, generate contradictory discussions within the 1 week waiting period, the arguments will be evaluated by a third researcher in the field (requested via email) and a decision by the new reviewer will be made, with discussion and reasoning posted on the Issue Tracker page. If the reviewers cannot agree, and a long term solution cannot be agreed upon, the issue will be addressed in a session at the next International Planarian Meeting. Following and participating in the discussions on proposed changes to PLANA is open to all, and only requires users to sign up for a free GitHub account and "watch" the PLANA repository. Watchers will receive email notifications when issues and discussions arise and when PLANA is updated."

We wholeheartedly agree with Reviewer 3 that further clarification of stem cell and progenitor cell names is a contentious issue that needs to be addressed by the field at large. The term 'neoblast', the most-often used planarian anatomical term in our Literature Search (Table S1), is over-determined: it refers to any cycling somatic stem or progenitor cell in an adult animal. Researchers use this term loosely and define it to suit their needs. It's problematic, but questions surrounding stem cell potential and plasticity are hardly settled science. PLANA attempts to capture some of this ambiguity and multiplicity, as it currently stands in the publication record, with the idea that class names, definitions, and lineage relationships can be edited and expanded upon, with community discussion, in the future. In its current form, PLANA includes the umbrella term 'neoblast', which by definition encompasses both pluripotent adult somatic stem cells and lineage-primed progenitor cells, as well as subclasses (e.g., 'clonogenic neoblast', 'zeta neoblast') that are defined operationally or molecularly. To promote ontology interoperability, we also imported general terms from other ontologies, like 'stem cell' (imported from the Cell Ontology) and 'germline stem cell' (imported from the Drosophila anatomy ontology). These general terms represent "parent terms" in the PLANA hierarchy, and they are used to organize an expanding collection of "child terms" through the "is a" relationship. For example, 'oogonial stem cell' is a female germline stem cell' is a 'germline stem cell' is a 'stem cell.'

© 2021. Published by The Company of Biologists under the terms of the Creative Commons Attribution License (https://creativecommons.org/licenses/by/4.0/).

We created the generic term 'progenitor cell,' an umbrella term that may refer to either a specialized neoblast or to a post-mitotic cell committed to a particular differentiation program. The definition is meant to capture the ambiguity regarding when lineage commitment occurs within the mitotic population - we don't yet know whether cells expressing *piwi-1* and a lineage-dedicated developmental transcription factor are irreversibly committed to differentiate. The definition also captures the insufficiency of language currently used in the field to demarcate cell types from cell states, as well as cases where researchers use the same term (e.g., 'pharynx progenitor cell') to refer to a neoblast as well as a post-mitotic cell that isn't yet terminally differentiated. We anticipate that this active area of research will yield new published findings that will be the basis for making PLANA revisions in the future, and we look forward to being active participants in these discussions.

5. Describe by way of at least one case study, how these tools that provide anatomy-based gene expression data compare to other methods (eg. single cell sequencing). These analyses could replace the superficial descriptions in Figure 6 and 7.

PLANA is not a standalone discovery tool, rather it is a framework used by other tools that facilitate collection, aggregation, and curation of experimental data. PLANA can be incorporated into a workflow to organize and annotate gene expression data, as we have proven with the creation of PAGE. Using the PAGE database, researchers can readily determine whether transcripts of interest in a given scRNA-Seq cluster, or across scRNA-Seq experiments analyzed using different transcriptomes, have been validated using WISH or FISH. Neither PLANA nor PAGE contain quantitative gene expression data, nor do they analyze primary data. Therefore, in our view, such a comparison is not appropriate or warranted.

We better highlight the organizing and synergistic function of PLANA in the text in the following lines (494-498):
"PLANA addresses two critical needs in our field: 1) PLANA provides a controlled anatomical vocabulary based on current field use. 2) PLANA is an open-source, adaptable, extensible, framework module that researchers can use to create new tools for universal and standardized data organization and aggregation, thus promoting searchability within and among large data sets."

6. Rewrite the manuscript to emphasize the value and application of this resource. Simplifying description of relationship terms will free-up a lot of space (Table 2 is more than sufficient). Figures 3 and 5 can also be removed to the supplement.

We thank Reviewer 3 for their manuscript editing suggestions. We made large scale changes to the manuscript to highlight that PLANA is framework to build other tools that require organization of information related to anatomy, not a replacement of current experimental discovery methods. We made the text more accessible to biologists in the field (see Reviewer 2 comments and rebuttal) and simplified the description of relationship terms. Specifically, we cut much of the detail surrounding use of the object properties (previous draft, lines 213-297) and now provide a simplified description in the main text (new draft, lines 238-254) and in Table 2, as the reviewer suggested.

References

Grohme, M. A., Schloissnig, S., Rozanski, A., Pippel, M., Young, G. R., Winkler, S., Brandl, H., Henry, I., Dahl, A., Powell, S., et al. (2018). The genome of Schmidtea mediterranea and the evolution of core cellular mechanisms. Nature 554, 56-61.
Wilkinson, M. D., Dumontier, M., Aalbersberg, I. J. J., Appleton, G., Axton, M., Baak, A., Blomberg, N., Boiten, J.-W., da Silva Santos, L. B., Bourne, P. E., et al. (2016). The FAIR Guiding Principles for scientific data management and stewardship. Sci Data 3, 160018.

Second decision letter

MS ID#: DEVELOP/2020/196097

© 2021. Published by The Company of Biologists under the terms of the Creative Commons Attribution License (https://creativecommons.org/licenses/by/4.0/).

MS TITLE: The Planarian Anatomy Ontology: A resource to connect data within and across experimental platforms

AUTHORS: Alejandro Sánchez Alvarado, Stephanie Nowotarski, Erin Davies, Sofia Robb, Eric Ross, Nicolas Matentzoglu, Viraj Doddihal, Mol Mir, and Melainia McClain

My apologies for the delay. I have now received all of the referees' reports on your revised manuscript, and have reached a decision. The referees' comments are appended below, or you can access them online: please go to BenchPress and click on the 'Manuscripts with Decisions' queue in the Author Area.

Reviewers 1 and 2 are satisfied with your revisions and recommend publication of your resource. You will see that Reviewer 3 appreciates the effort and potential value of your work, but also expresses concerns regarding the functionality of your resource based on sample searches. I am aware that this ontology is intended to be a living resource, to be tested and improved in response to feedback from community users. Yet, some explanation or modification is needed to address how Reviewer 3 was unable to capture informative data with several of their inputs, and to help ensure that most searches are fruitful at ultimate launch. Though it is unusual to have more than one round of major revision for Development, I feel it is best for your manuscript and the ultimate reception of the resource to address these concerns, which may involve additional modifications of the online tool.

Please attend to Reviewer 3's comments and ensure that you highlight all changes made in the revised manuscript. It is also important to provide a point-by-point response detailing how you have dealt with the each of the reviewer comments in the 'Response to Reviewers' box. If you do not agree with any of their criticisms or suggestions please explain clearly why this is so.

Reviewer 1

Advance summary and potential significance to field

This paper presents an anatomical ontology for planarians based on literature review and integration of existing ontologys. This is demonstrated in part in the construction of an expression database using the ontology. The paper presents a convincing picture that the project is thorough and can make a significant contribution within the field.

Comments for the author

Revisions addressed all of my concerns with the original manuscript making strategic decisions in building the atlas clearer.

Reviewer 2

Advance summary and potential significance to field

This manuscript reports an ontology that will facilitate the future development of the planarian model system.

Comments for the author

The authors have put substantial effort into the revision of the manuscript.
Overall, our comments have been adequately addressed. But we would like to encourage the authors to facilitate the parsing of the source literature for specific terms in future renditions of the database.

Reviewer 3

Advance summary and potential significance to field

© 2021. Published by The Company of Biologists under the terms of the Creative Commons Attribution License (https://creativecommons.org/licenses/by/4.0/).

The authors have created an online tool (The Planarian Anatomy Ontology) and claim two key contributions:

1) that this ontology serves as a good example to others particularly in developing model organisms that may be interested in building their own ontologies following best practices, and 2) that PLANA together with PAGE and Rosetta Stone provide tools to researches that allow them to "curate, aggregate, and mine data" from existing planarian publications up to 2019. In regards to the former, I generally agree that the authors have succeeded though I defer to the other reviewers as this is not an area within my expertise. With regards to the latter, I have tried to replicate the "end-user" experience of using these tools and I have found it to often be frustrating (examples below). I was previously unconvinced that these resources provide significant utility to the field and tried to suggest that the authors provide more emphasis on the value and application of these tools above the superficial examples provided in the paper. Additionally, I suggested that the authors could highlight how their ontology helps clarify existing conflicts/inconsistencies in the use of terminology among planarian biologists. The authors acknowledge that these are difficult problems and that PLANA is meant to be a "living resource" that is open to the community to debate and make suggestions, and they side-step my call for them to demonstrate more thoroughly the significance of these resources. I appreciate the enormous efforts that have gone into producing these tools, but I am left once again with the impression that its significance is limited. This is further compounded but the fact that trying to use these tools, in my experience, is error prone. I think most researchers who might try to use these tools will be suspicious of its reliability.

Comments for the author

The authors have created a number of videos to guide researches in using PLANA and PAGE. These are indeed very helpful and I commend the effort (although I do recommend that the authors edit them to correctly pronounce Schmidtea mediterranea). However, when I tried to replicate one of the tasks described in a video I immediately encountered problems:
PAGE can be mined using terms, transcripts, or publications. In one of the videos, the lead author gives the example that a researcher could take a gene name from a planarian publication and search PAGE to identify all other instances in which this gene has been described in the literature. The example that was used was sp6-9. I replicated this and found the same results. I then pulled-up a number of planarian papers and searched for genes that were published. The following genes returned no results:
tropomyosin, nanos, TSPAN-1, wnt1, ptk7, nr4A, FoxA

I did however succeed in getting results for wntP-2 and notum.

I cannot explain why the searches failed; some of these gene names are even in the title of papers and some represent canonical, well known markers of specific cell types. Moreover, expression patterns for wnt1, ptk7 FoxA, wntP-2, and notum are all described in the same figure in Lander and Petersen, Elife (2016). These genes are certainly described elsewhere but it is confusing to me how this could happen since the curation was done manually by publication. In situ patterns for all these genes are in the same figure and they were used as markers for different anatomical regions. How did some but not all of these genes make it into the database?

I then looked-up the Pubmed ID of this paper (27074666) and used the search by publication tool in PAGE.

Only 6 results were found and they did not include most of the genes in the paper. This showed me that the criteria the curators used to describe at least in situ patterns is clearly lacking. Now to my surprise, Ptk7 was one of the hits. So I returned to the search by transcript tool and indeed, capitalizing "P" succeeded in returning results. However, capitalizing "W" for Wnt1 and using lower case "f" for foxA did not remedy the problem. In any case, it is a flaw that the use of upper or lower case affects these search results at all. And the lack of results for such famous markers certainly dims my confidence in this tool.
It seems like this site is not yet ready for launch and there are fundamental errors, which do not allow for the execution of the tasks described in the videos.

© 2021. Published by The Company of Biologists under the terms of the Creative Commons Attribution License (https://creativecommons.org/licenses/by/4.0/).

Second revision

Response to Reviewers

We thank all of the reviewers for their comments and constructive criticism of PLANA, PAGE, and the planosphere website. All three reviewers agreed that the main tool unveiled in our manuscript, PLANA, is well-described and executed, and concur that our description of PLANA's construction serves as a model whereby other emerging research organism communities can build their own anatomy ontologies according to best practices. This was our primary goal for this publication. In addition, we present applications of PLANA to demonstrate the utility of our ontology for data curation, aggregation, and mining across experimental platforms. We thank Reviewer 3 for their diligent testing of the Planarian Gene Expression Database (PAGE), itself a living resource that is editable and extensible, and agreed that there were three points that needed to be addressed: 1) "completeness" of the manual curations comprising the PAGE database, 2) consistency of PAGE returns, and 3) assumptions about how the PAGE search works.

To address completeness of the PAGE database we conducted an audit of our curations. We picked 10% of the curated papers in PAGE at random and asked a second curator to independently annotate the paper. When we compared both rounds of annotation we found that human variability accounted for differences in annotation and these variables could be broken down into three clear categories:

A. **Differences in the choice of PLANA term used by a curator.** Different curators sometimes chose closely related but different PLANA terms. For example, one curator annotated transcript expression in 'parenchymal cell' while a second curator assigned the same set of transcripts to 'parenchyma.' Similarly, we found an instance where the same set of transcripts was annotated as expressed in a 'reproductive organ' and 'reproductive structure' by two different curators. In both of these cases, the publications contained both terms in their text, suggesting the curators selected different author-assigned annotations. PLANA has two features that reconcile these differences. First PLANA's association of synonyms with terms accommodates variations in used equivalent language. Second, PLANA's transitivity, engendered through relationship terms, facilitates broader search returns. In the examples above, PAGE searches for transcripts that are **part of** 'parenchyma' will also return transcripts annotated as expressed in 'parenchymal cell,' since 'parenchymal cell' is **part of** 'parenchyma.' Similarly, searching for transcripts that are **part of** the 'reproductive system' will return transcripts associated with expression in both 'reproductive structure' and 'reproductive organ'.

B. **Differences in the publication ID for a transcript.** Some publications do not provide accession numbers or transcript IDs, particularly for transcripts that were previously described in a publication record. In these cases, curators attempted to associate a named gene with a Genbank accession number, transcript ID, EST, primary sequence, or primer sequences in referenced publication(s). EST, primary sequence, or primer sequences were used to identify a reference transcript ID in the smed20140614 transcriptome via BLASTN. In some cases, data is lost - i.e., expression data cannot be annotated - because reference sequences cannot be identified. In other instances, curators identified different sequence IDs from cited publications or input a smed20140614 transcriptome ID directly into the PAGE database. By design, PAGE users do not encounter this variability in curation since the PAGE output is mapped to the smed20140614 transcriptome. We updated language in the tutorial video (https://planosphere.stowers.org/search/page/transcript - "About this search") to communicate that PAGE searches by transcript ID and or accession are the most direct, reliable, and inclusive search method in contrast to gene names. We also changed the language on the PAGE overview video (https://planosphere.stowers.org/search/page/about) to make this point clear. The PAGE search by transcript webpage now includes the following point under "Search Tips": "Using transcript IDs or accessions such as JQ425152, SMED30008505,

© 2021. Published by The Company of Biologists under the terms of the Creative Commons Attribution License (https://creativecommons.org/licenses/by/4.0/).

dd_Smed_v4_1757_0_1 produce more comprehensive results than using gene names such as cintillo."

C. **Missed Annotations**

i. **Change in curation rules over time**. Rules for PAGE curation evolved during the curation period as ambiguous and challenging examples were encountered, and as training through experience led curators to adopt standardized practices for annotation. Fortunately, PAGE is a living resource and revision of existing entries and addition of new annotations is possible, and indeed encouraged. To facilitate updates and additions to PAGE, we have included the PAGE curation rules, included in supplemental table 16. Our manuscript references this addition in the main text (line 593- 594): " Rules for curation of literature for PAGE can be found in the supplemental table 16."

ii. **Missing curations.** Curator-driven differences in annotation and omissions in the annotation record can both account for missing information in the PAGE database. As discussed previously, some level of subjectivity and variation is inherent and due to inconsistencies in language and reporting of expression data and/or transcript/sequence information by authors. Human error is also undoubtedly a factor, as Reviewer 3 correctly pointed out regarding PMID: 27074666. During our audit we found two other instances from twenty publications where expression data from supplemental tables were missing from PAGE (PMID:27034770 and 21282632). In all three noted cases, annotations for missing data were added to the PAGE database.

To facilitate additions to PAGE in accordance with the PAGE curation rules we have added an issue tracker page in the GitHub repository for PAGE where users can report errors (https://github.com/planosphere/PAGE/issues). This is noted in the revised manuscript, lines 594-596: " PAGE has an accompanying issue tracker for requested changes or additions to curations found at https://github.com/planosphere/PAGE/issues (Table S13)." We also added links to the PAGE issue tracker on the PAGE overview webpage (https://planosphere.stowers.org/search/page/about - "How to Contribute" section) on Planosphere, and a webpage outlining the rules for PAGE curation (https://github.com/planosphere/PAGE/blob/master/curation_rules.md).

The uncovered inter-curator variation was expected, as this is a subjective process due to variation in choice of language and available expression information in the publications themselves. Widespread buy-in and adoption of a standardized vocabulary, provided by PLANA, in their workflows and manuscripts will help to minimize some of this variation in the future. PAGE is a resource that provides a quick overview of existing information and is a starting point for in-depth exploration of the underlying data, much like GO analysis would suggest putative functions for a given gene. Neither PAGE nor GO, or any overview tool, should replace in-depth analysis and experimental testing and confirmation of an initial hypothesis. We make this clear in the following passage in the revised manuscript, lines 425-426: "Aside from providing individual records of expression, PAGE provides an initial platform to survey the expression landscape."

With respect to the concerns Reviewer 3 made with respect to the PAGE search function, we have done the following:

1. To ensure capitalization differences do not interfere with search results we altered both our PAGE transcript search and the Rosetta Stone Transcript Mapper to be case-insensitive.
2. We re-filmed the PAGE overview tutorial to state the search works best with either the original transcript IDs or accessions. Transcript ID, especially smed20140614 can be identified using the Rosetta stone transcript mapper or the BLAST search. We added the caveat that folks **can** search with colloquial names, but this will not identify all transcripts and their associated annotations. Our search-by-name functions on par with Planmine, where one cannot reliably search by all published gene names (e.g., cintillo). To improve on the search-by-name and to mirror Planmine's functionality we incorporated the ability

© 2021. Published by The Company of Biologists under the terms of the Creative Commons Attribution License (https://creativecommons.org/licenses/by/4.0/).

to search by the gene symbols associated with transcripts from Planmine. Now the PAGE search-by-transcript function works when the input is a gene symbol found in Planmine (e.g., WNT1).

Third decision letter

MS ID#: DEVELOP/2020/196097

MS TITLE: The Planarian Anatomy Ontology: A resource to connect data within and across experimental platforms

AUTHORS: Alejandro Sánchez Alvarado, Stephanie Nowotarski, Erin Davies, Sofia Robb, Eric Ross, Nicolas Matentzoglu, Viraj Doddihal, Mol Mir, and Melainia McClain

I have received the final referee report on the above manuscript, and have reached a decision. The referee comments are appended below, or you can access them online: please go to BenchPressand click on the 'Manuscripts with Decisions' queue in the Author Area.

As we discussed by phone, the remaining reviewer expresses concerns; however, based on the positive nature of the other reviews and the feeling that this work will provide a potentially valuable community resource, we would like to publish the resource in Development. I ask that you take one additional opportunity to consider the reviewer comments and revise your resource and its description to ensure that it is in the form you feel will be best received, before submitting a final version of the work for publication. When you do submit the work, please summarize in a cover letter any revisions you have made.

Reviewer 3

Advance summary and potential significance to field

Overall, this resource is extremely limited in its scope and functionality. The ontology database establishes relationships such as “a neuron is in the brain which is in the head”. On its own, this is not very useful but coupled with gene expression data, I was interested to see how this resource could provide added value to researchers. The authors have decided against using this database to clarify inconsistent terminology (such as stem cells vs. progenitors). The authors have decided against curating by gene name to reconcile multiple transcript IDs that refer to the same genes when providing gene expression readouts of each anatomical term, in this curation of 200 papers. Given this, I am forced to conclude that the added value of this resource is limited.

Comments for the author

Response to Part B:
This is extremely disappointing. If this tool was going to be useful, it would be because the authors have devised a system to resolve the problem of multiple transcript IDs referring to the same gene in a streamlined search feature.

Searching by exact transcript ID seriously limits the usefulness of this tool.
I decided to use an easy test case- FoxA, a canonical pharynx marker published from these authors' lab: “Selective amputation of the pharynx identifies a FoxA-dependent regeneration program in planaria. Elife. 2014 Apr 15;3:e02238. doi: 10.7554/eLife.02238. PMID: 24737865”

A search for all transcripts expressed in the pharynx produced 3227 results.
When I searched for “FoxA” within those results there were no hits. This is because I needed to search for “forkhead box A-1”, which produced 10 hits representing multiple transcripts IDs, publications, type of experiment etc.

© 2021. Published by The Company of Biologists under the terms of the Creative Commons Attribution License (https://creativecommons.org/licenses/by/4.0/).

In this rebuttal, the authors state that the criteria they set for themselves was to only use gene names that were already annotated in Planmine and FoxA is not one of them. This explains the failed search. So, I looked for the Planmine transcript ID and found dd_Smed_v6_10718_0_1. To be sure that this transcript ID was included in PLANA/PAGE, I used the "Search by transcript" tool and it returned 61 results including: expressed in "pharynx", " pharynx progenitor cell", "embryonic pharynx", "parapharyngeal region" etc.

I returned to the 3227 results for transcripts expressed in pharynx and searched dd_Smed_v6_10718_0_1: no results. So even though this transcript ID is identified as a transcript expressed in the pharynx, a search for all transcripts expressed in the pharynx does not recognize the fact. I assume that this is because during the annotation, other transcript IDs were matched with individual publications.

This is a weakness of relying on transcript IDs, since multiple IDs refer to the same gene.
This was the first and only search I preformed to test the platform as I considered it to be an easy and straightforward test case. Thus, these results are disappointing.
Response to Part Cii
Since in my last review I chose PMID: 27074666 at random, I was extremely surprised that, according to this comment, I happen to have chosen one of only three publications with missing curations identified in their audit (and it seems the only publication in which the missing curations were in the main text of the figure). So, I went back to the paper above about FoxA (PMID 24737865). I used the "Search by publication" tool and found that the only results were for "Forkhead box A-1". Figure 6 of this paper shows expression patterns for laminin, npp-1 porcupine, ndk, PC2 and collagen. It is unclear to me what criteria the curators used to disregard these or why they were not assigned a smed20140614 transcriptome ID, as stated in this rebuttal.

Response to authors' comments on my previous concerns:
1. Thank you for removing case-sensitivity, even though search by gene names is now not recommended.
2. If users are expected to search by exact transcripts IDs, what is the point?
If I already knew all the exact transcript IDs used in the planarian literature for say, FoxA, then I already know of all the relevant papers. If the expectation is that the user must first identify all redundant transcript IDs using Rosetta Stone for each gene of interest, then comb through all the duplicated search results for each transcript ID, this resource is meant for a very small number of users in very limited circumstances.

Third revision

<u>Author response to reviewers' comments</u>

We appreciate Reviewer Three's feedback regarding returns from the PAGE database and have undertaken steps to ensure that PAGE will continue to be updated and expanded with input from members of the scientific community. We created a Github Issue Tracker (https://github.com/planosphere/PAGE/issues/new/choose) and clearly describe the procedures for adding or editing existing annotations in the PAGE database (https://github.com/planosphere/PAGE/blob/master/curation_rules.md). Using this process, we rectified the missing data described by Reviewer Three from PMID 16311336.

We also added language to the PAGE search instructions to educate users about what can be expected for search returns. As part of the rollout for our publication, we will extend a planarian field-wide invitation to source volunteer curators for PLANA and PAGE at the continuing virtual "Flatworm Friday" series this fall. We will provide demonstrations and how-to guides for authors' to contribute annotations from their manuscripts to PLANA and PAGE. Continued updates and curation by authors and stakeholders in the community will ensure that PLANA and PAGE evolves in accordance with usage and needs of the field over time. We appreciate the constructive criticism and feedback that you and the review team have afforded our work, and are pleased with the

© 2021. Published by The Company of Biologists under the terms of the Creative Commons Attribution License (https://creativecommons.org/licenses/by/4.0/).

improved manuscript and your editorial decision. Thank you again for appreciating the value of sustained investment in community resources for emerging developmental models.

Fourth decision letter

MS ID#: DEVELOP/2020/196097

MS TITLE: The Planarian Anatomy Ontology: A resource to connect data within and across experimental platforms

AUTHORS: Alejandro Sánchez Alvarado, Stephanie Nowotarski, Erin Davies, Sofia Robb, Eric Ross, Nicolas Matentzoglu, Viraj Doddihal, Mol Mir, and Melainia McClain

ARTICLE TYPE: Techniques and Resources Article

I am happy to tell you that your manuscript has been accepted for publication in Development, pending our standard ethics checks.

© 2021. Published by The Company of Biologists under the terms of the Creative Commons Attribution License (https://creativecommons.org/licenses/by/4.0/).
